# Increasing the Diameter of Vertically Aligned, Hexagonally Ordered Pores in Mesoporous Silica Thin Films

**DOI:** 10.1021/acs.langmuir.1c02854

**Published:** 2022-02-08

**Authors:** Nabil A. N. Mohamed, Yisong Han, Andrew L. Hector, Anthony R. Houghton, Elwin Hunter-Sellars, Gillian Reid, Daryl R. Williams, Wenjian Zhang

**Affiliations:** † School of Chemistry, University of Southampton, Highfield, Southampton SO17 1BJ, U.K.; ‡ Department of Physics, University of Warwick, Coventry CV4 7AL, U.K.; § Department of Chemical Engineering, Imperial College London, London SW7 2AZ, U.K.

## Abstract

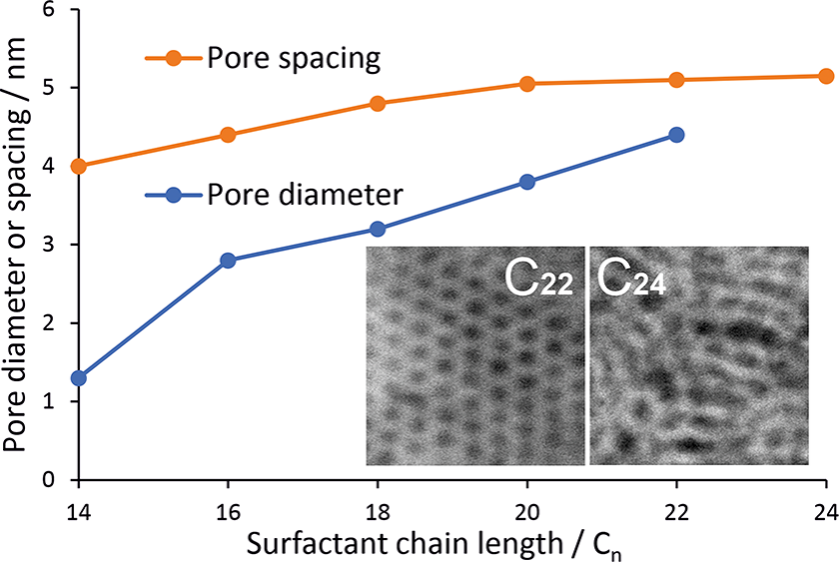

The variation in pore size in mesoporous films produced by electrochemically assisted self-assembly (EASA) with the surfactant chain length is described. EASA produces a hexagonal array of pores perpendicular to the substrate surface by using an applied potential to organize cationic surfactants and the resultant current to drive condensation in a silica sol. Here, we show that a range of pore sizes between 2 and 5 nm in diameter is available with surfactants of the form [Me_3_NC_*n*_H_2*n*+1_]Br, with alkyl chain lengths between C_14_ and C_24_. The film quality, pore order, pore size, and pore accessibility are probed with a range of techniques.

## Introduction

Oriented mesoporous silica films are potential hard templates for the electrodeposition of nanowire-based devices,^1^ with possible applications in Li-ion batteries,^2,3^ thermoelectrics,^4^ and supercapacitors.^5^ In order for the electrode surface to remain accessible to the electrolyte it is necessary to arrange the pores perpendicular to the substrate surface, often referred to as the vertical orientation. Although the growth of platinum nanowires in silica with horizontal pores occurred readily, Kanno et al.^6^ showed that the electrodeposition of Au nanowires was much easier in perpendicular pores.^7^ While the literature on electrodeposition in very small pore templates is limited, there are some examples, for example, of 3 nm Sn nanowires^8^ or 5 to 8 nm bismuth nanowires,^9^ produced in this way.

Pore orientation, pore diameter, and organo-functionalization^10,11^ are important factors in the rate of ion diffusion through porous films. Etienne and co-workers reported TiO_2_ with the anatase structure in an open porous network, in which positively charged species were more mobile than negatively charged ones.^12^ The fine tuning of parameters has made silica films attractive materials for biosensing and electrochemical sensing applications.^13,14^ For example, Nasir et al.^15^ developed an electrochemical pathway for detecting cationic paraquat salts in oriented mesoporous silica films.

The most common approach to preparing mesoporous silica films is using evaporation-induced self-assembly (EISA).^16−18^ This method involves the preparation of an aqueous ethanol solution containing a surfactant and silica precursors to produce a homogenous solution of stable species. The initial surfactant concentration of the prepared solution is significantly lower in comparison to the critical micelle concentration (*C*_o_ ≪ CMC). Once the films have been deposited in solution by either dip or spin coating, the ethanol solvent evaporates from the deposited film, resulting in the surfactant concentration increasing, which in turn triggers the self-assembly process and micelle formation, producing well-organized porous structures in the liquid crystalline phase before condensing the silica species. EISA tends to produce ordered silica films with the pores either horizontal to the substrate surface or randomly oriented.

Several strategies have been devised to produce orientated silica films through the application of a magnetic field,^19^ epitaxial growth,^20^ and radio sputtering.^21^ For example, Yamauchi used an external magnetic field to control pore alignment from an EISA process either vertically or horizontally to the substrate surface.^22^ Otomo et al.^23^ synthesized aligned mesoporous silica films by a radiofrequency sputter deposition of Co–Si–O films where the surface morphology was influenced by a range of argon gas pressures followed by wet chemical etching to remove the Co fragment from the films. Stöber-solution growth is based on the formation of hemispherical micelles of a cationic surfactant on a negatively charged substrate and interactions with silica oligomers that encourage the vertical growth of those micelles.^24^ This method can take days to grow very thin, aligned films.

Walcarius and co-workers^25^ revealed that the preparation of vertically oriented mesoporous silica films was possible under electrochemical control. The mechanism for the electrochemically assisted self-assembly (EASA) of silica comprises of applying a negative potential to the substrate to be coated while it sits in a solution containing the surfactant and silica precursors dissolved in an ethanol/water mixture. This results in the generation of hydroxide ions in the solution, and the pH swings from acidic to alkaline at the electrode surface. The electric field is thought to play a pivotal role in the self-organization of the cationic surfactant into hemimicelles, and the pH change catalyzes the polycondensation of the sol, leading to mesoporous silica films with perpendicular (vertical) pore channels (2–3 nm) and uniform film thickness (150–200 nm). Mesoporous films can be obtained using the EASA method within as little as 20 s.

EASA was developed with a [Me_3_NC_16_H_33_]Br (CTAB or C_16_TAB) surfactant, and this leads to pores with a diameter of around 2 nm depending on conditions and substrates.^26^ Robertson et al. showed that mesitylene can be used as a swelling agent for CTAB-templated EASA films on TiN, causing the pore diameter to increase from 1.6 to 2.4 nm.^27^ However, larger still amounts of mesitylene resulted in a reduction in the order of the porosity. Larger vertically aligned pores may facilitate the use of such films as hard templates for nanowire electrodeposition and increase the range of sensing applications. Ullah et al. recently showed that polyaniline nanowires with different widths could be produced in EASA silica films produced with C_16_TAB or C_18_TAB.^28^ Herein, we demonstrate that systematic increases in the pore diameter beyond 2 nm are achievable by EASA when utilizing surfactants created by further extending the single straight alkyl chain.

## Experimental Section

Tetradecyltrimethylammonium bromide (C_14_TAB, 99%), hexadecyltrimethylammonium bromide (C_16_TAB, 98%), and octadecyltrimethylammonium bromide (C_18_TAB, 98%) were purchased from Sigma-Aldrich. The ^1^H and ^13^C{^1^H} nuclear magnetic resonance (NMR) data for the surfactants in *d*-chloroform were collected using a Bruker AVII400 spectrometer at 25 °C. Solutions for mass spectrometry were prepared by dissolving 50 μg of the surfactant in 1 mL of methanol. The surfactants were analyzed using ultrahigh performance liquid chromatography coupled to a TQD mass tandem quadrupole mass spectrometer (Waters) using a TUV detector at 254 nm. The chromatography analysis was carried out using a Waters BEH C18 column (50 mm × 2.1 mm, 1.7 μm) with a flow rate of 0.6 mL/min. The mobile phase was made up of 0.2% formic acid in an aqueous solution and 0.2% formic acid in acetonitrile. This was followed by the recording of the mass spectrum in the positive ion electrospray ionization mode.

**Scheme 1:**
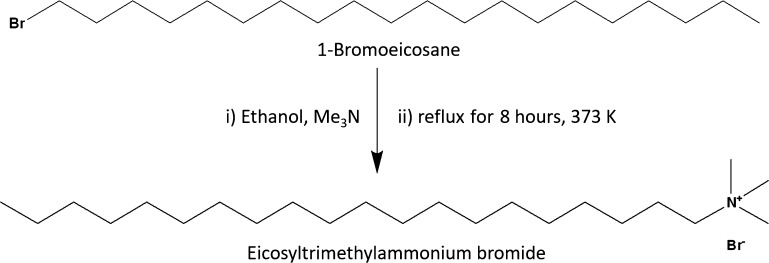
Reaction of 1-Bromoeicosane with Trimethylamine to Form the Quaternary Ammonium Salt, C_20_TAB; The Same General Method Was Used for C_22_TAB and C_24_TAB Surfactants

The synthesis of longer chain alkyltrimethylammonium bromide surfactants (Scheme 1) was carried out according to Scheraga et al.^29^ The synthesis used alkyl bromides C_*n*_H_2*n*+1_Br where *n* is equal to 20 (5.0001 g, 13.83 mmol ≥ 97%, Sigma-Aldrich), 22 (5.0001 g, 12.84 mmol, 96%, Santa Cruz Biotechnology), or 24 (0.8915 g, 2.14 mmol, synthesized as described below). The alkyl bromide was dissolved in ethanol (25 cm^3^) and 30% trimethylamine in an ethanol solution (7.6000 g, 128.6 mmol for C_20_ and C_22_, or 1.3000 g, 22.0 mmol for C_24_, Sigma-Aldrich) was added dropwise into this solution. The mixture was refluxed at 100 °C for 8 h with stirring under a dry ice condenser. The solution was then filtered and stored overnight in a freezer to precipitate the product. The crude product was filtered, and the resulting solid was recovered using a rotary evaporator, then recrystallized three times from ethanol. The final yields were 4.50 g, 10.7 mmol eicosyltrimethylammonium bromide (C_20_TAB, 90%, off white solid), 4.22 g, 9.4 mmol docosyltrimethylammonium bromide (C_22_TAB, 84%, white solid), and 0.78 g, 1.6 mmol tetracosyltrimethylammonium bromide (C_24_TAB, 88%, white solid).

The synthesis of 1-bromotetracosane (C_24_H_49_Br) was based on the procedure reported by Al-Dulayymi et al.^30^ The method involved dissolving triphenylphosphine (0.800 g, 3.05 mmol, 99%, Avocado Research Chemicals Ltd.), tetracosan-1-ol (1.000 g, 2.82 mmol, >98%, Tokyo Chemical Industry UK Ltd.), and *N*-bromosuccinimide (1.500 g, 8.43 mmol, 99%, Sigma-Aldrich) in dry dichloromethane (50 cm^3^). The reaction mixture was stirred in a water bath at an ambient temperature for 5 h and then quenched with water (200 cm^3^), extracted into dichloromethane and dried using a rotary evaporator to give a red solid. The product was then refluxed in petroleum ether (500 cm^3^) and ethyl acetate (5 cm^3^), filtered, and then the filtrate was dried using a rotary evaporator to give C_24_H_49_Br (0.892 g, 2.14 mmol, 89.2% yield). The material (C_24_H_49_Br) produced was used directly for the preparation of the C_24_TAB surfactant. NMR and mass spectra are shown in the Supporting Information (Figures S1–S9).

### C_20_TAB

^1^H NMR (CDCl_3_): δ/ppm 0.83 (t, CH_3_, [3H]), 1.17 (br s, CH_2_, [32H]), 1.26–1.37 (m, CH_2_, [2H]), 1.63–1.77 (m, CH_2_, [2H]), 3.41 (s, CH_3_, [9H]), 3.47–3.56 (m, CH_2_, [2H]). ^13^C{^1^H} NMR (CDCl_3_): δ/ppm 14.12, 22.70, 23.23, 26.19, 26.22, 29.23, 29.32, 29.36, 29.47, 29.54, 29.59, 29.63, 29.66, 29.68, 29.70, 29.71, 31.92, 53.41, 58.43, 67.12. MS (ESI^+^ in CH_3_OH): found *m*/*z*, 340.58; required for {C_23_H_50_N^+^} *m*/*z*, 340.

### C_22_TAB

^1^H NMR (CDCl_3_): δ/ppm 0.82 (t, CH_3_, [3H]), 1.19 (br s, CH_2_, [36H]), 1.29–1.37 (m, CH_2_, [2H]), 1.61–1.65 (m, CH_2_, [2H]), 3.41 (s, CH_3_, [9H]), 3.47–3.53 (m, CH_2_, [2H]). ^13^C{^1^H} NMR (CDCl_3_): δ/ppm 14.10, 22.72, 23.21, 26.17, 29.22, 29.27, 29.32, 29.35, 29.36, 29.46, 29.59, 29.65, 29.66, 29.69, 29.71, 31.89, 31.91, 31.93, 42.63, 53.45, 67.18, 67.21. MS (ESI^+^ in CH_3_OH): found *m*/*z*, 368.60; required for {C_25_H_54_N^+^} *m*/*z*, 368.

### C_24_TAB

^1^H NMR (CDCl_3_): δ/ppm 0.82 (t, CH_3_, [3H]), 1.18 (s, CH_2_, [40H]), 1.22–1.33 (m, CH_2_, [42H]), 1.63–1.72 (m, CH_2_, [2H]), 3.40 (s, CH_3_, [9H]), 3.46–3.54 (m, CH_2_, [2H]). ^13^C{^1^H} NMR (CDCl_3_): δ/ppm 14.12, 22.70, 23.25, 25.32, 25.33, 26.18, 29.20, 29.29, 29.33, 29.37, 29.44, 29.55, 29.58, 29.65, 29.67, 29.69, 29.71, 31.94, 43.65, 53.46, 53.51, 53.54, 53.59, 53.60. MS (ESI^+^ in CH_3_OH): found *m*/*z*, 396.70; required for {C_27_H_58_N^+^} *m*/*z*, 396.

### Mesoporous Silica Growth

#### C_14_TAB–C_18_TAB

The synthesis of vertically ordered silica films under electrochemical control used the method previously reported by Goux et al.^31^ The electrolyte was prepared by mixing C_14_TAB (0.4801 g, 1.43 mmol), C_16_TAB (0.4801 g, 1.32 mmol), or C_18_TAB (0.4801 g, 1.22 mmol) into 0.1 mol dm^–3^ sodium nitrate (NaNO_3_) in 20 cm^3^ water and 20 cm^3^ ethanol, then tetraethylorthosilicate (TEOS, 98%, Sigma-Aldrich, 905 μL, 4.08 mmol) was added to the solution. The sol was adjusted close to pH 3 using 0.2 mol dm^–3^ HCl and allowed to hydrolyze for a duration of 2.5 h. The ratio for the surfactant:silica precursors was kept constant at around 0.3 with each experiment containing a newly made up sol electrolyte.

#### C_20_TAB–C_24_TAB

The sol preparation was altered slightly when using C_20_TAB (0.3000 g, 0.71 mmol), C_22_TAB (0.3000 g, 0.67 mmol), and C_24_TAB (0.3000 g, 0.63 mmol) due to surfactant solubility problems with the standard method described above. The electrolytes were prepared by mixing 0.1 mol dm^–3^ NaNO_3_ in water (20 cm^3^) with isopropyl alcohol (20 cm^3^), adding TEOS (905 μL, 4.08 mmol), and adjusting the sol pH to between 3 and 3.5 using 0.2 M HCl. The sol was then stirred for 90 min. The surfactants were added to the sol and allowed to stir for a further 60 min. The C_22_TAB and C_24_TAB surfactants did not dissolve in the solution at 25 °C, so the temperature was raised to 35 °C, which caused dissolution. The solutions were then allowed to hydrolyze for 2.5 h. The surfactant:silica ratio was kept constant at around 0.17 with each experiment using a fresh sol electrolyte.

The electrodeposition process of porous silica was carried out in a Teflon cell with 2 × 1 cm indium tin oxide (ITO)-coated glass (surface resistivity of 8–12 Ω^–1^, Sigma-Aldrich) used as the working electrode. Prior to deposition the electrodes were washed with water and ethanol and dried under N_2_ gas. The working electrode was submerged vertically into the sol electrolyte, a stainless steel cone was used as the counter electrode and a silver rod was used as the reference electrode. A Biologic SP150 potentiostat was used to apply a constant potential of −1.25 V (vs Ag/Ag^+^) for a duration of 20 s. After each experiment the silica films (1 × 1 cm) were quickly rinsed with water and ethanol, then dried in an oven at 130 °C for 16 h. To remove the surfactants from the pore channels, the films were then immersed in a solution of 0.2 mol dm^–3^ HCl (Fischer Scientific) in ethanol for 15 min under gentle stirring.

### Film Characterization

Cyclic voltammetry (CV) was used to determine the accessibility of the pores in the mesoporous silica films on ITO electrodes using a range of redox probes. The aqueous probe solution was made up of 0.5 mmol dm^–3^ ferrocene methanol (FcMeOH), 5 mmol dm^–3^ hexaammineruthenium(III)chloride {[Ru(NH_3_)_6_]^3+^}, or 0.5 mmol dm^–3^ each of potassium hexacyanoferrate(III) {[Fe(CN)_6_]^3–^} and potassium hexacyanoferrate(II)trihydrate {[Fe(CN)_6_]^4–^}, in all cases with a 0.1 mol dm^–3^ NaNO_3_ supporting electrolyte. The working electrode was the ITO coated with mesoporous silica. A platinum gauze was used as the counter electrode, and the reference electrode contained Ag/AgCl in a 4 mol dm^–3^ KCl solution. The potentiostat used was a Biologic SP150.

A Rigaku Smartlab X-ray diffractometer with a Hypix-3000 2D detector was used for grazing incidence small angle X-ray scattering (GISAXS) experiments. The incidence angle for all samples was 0.25°, with measurements collected as two-dimensional (2D) images or as one-dimensional (1D) scans collected in-plane with 0.5° incident and 0.228° exit Soller slits and an angle range of 1–10° 2θ. Scanning electron microscopy (SEM) images were collected using a Jeol JSM-6500F operating at an accelerating voltage of 5 kV. To overcome charging, the porous silica films were coated with a thin layer of gold to improve the conductivity of the material before obtaining the top view and cross-sectional scanning electron micrographs. The scanning transmission electron microscopy images of the mesoporous silica films were recorded using a JEOL ARM200f double-corrected transmission electron microscope operated at 200 kV. Transmission electron microscopy (TEM) specimens were prepared by scraping the silica films off the substrate and suspending them on lacey carbon films. The TEM specimens were tilted during the TEM observations to allow for the establishment of an edge-on condition for some silica flakes (the pores directly facing the electron beam), when the size and the arrangements of the pores are clearly revealed.

Ellipsometric porosimetry (EP) experiments were performed using a dynamic vapor sorption instrument (Surface Measurement Systems Ltd., UK) coupled with a FS-1 multiwavelength ellipsometer (Film Sense, USA). The toluene vapor sorption experiments were carried out in an environmental chamber at a fixed temperature of 25 °C, at atmospheric pressure, and with a solvent partial pressure range between 0 and 95% *P*/*P*_0_. The partial pressure was maintained using mass-flow controllers operating in the closed-loop mode using dry air as the carrier gas with a flow rate of 100 mL/min and the partial pressure was monitored using a speed of sound sensor. The ellipsometer uses four wavelengths (465, 525, 595, and 635 nm) at an angle of incidence of approximately 65° to produce the highest possible signal intensity in the detector. The mesoporous silica films were exposed to toluene in fixed partial pressure steps ranging between 0.5 and 5% for 20 min per step to reach equilibrium, and the refractive index was continuously measured to produce isotherms.

## Results and Discussion

The EASA method was originally devised by Walcarius^25^ and has been shown to be a versatile method to produce highly ordered silica films with the pores vertical to the substrate. Previously, this process largely used C_16_TAB as the surfactant, resulting in ∼1.6 nm aligned, hexagonally ordered pores. Walcarius’ group^31^ studied the pore accessibility of EASA films grown with C_12_TAB to C_18_TAB using a FcMeOH redox probe. It was concluded that the increase in current from the CV plots was associated with increases in the lipophilic surfactant chain length.

In the present study, the deposition of silica films using a series of cationic surfactants with different alkyl chain lengths, C_14_–C_24_, was undertaken with the aim of producing larger pore diameters than currently available in vertical orientation.^26,32^ Deposition potentials above −1.2 V (vs Ag/Ag^+^) result in no film formation, whereas below −1.3 V (vs Ag/Ag^+^), the ITO electrode is damaged by the reduction of indium oxide. Depositions were carried out at −1.2 V. The range at which depositions occur is between 10 and 20 s, with thickness values of 40–150 nm, and with lengthier deposition times resulting in a loss of uniformity due to surface aggregates.^25,33^ The longer alkyl chain lengths increase the hydrophobicity of the surfactant, so the sol composition and temperature had to be varied to achieve surfactant solubility and produce highly ordered porous silica films, including switching the ethanol component of the sol to isopropyl alcohol. The films were then dried and the surfactant was removed by solvent extraction as described above.

### Structure and Characterization of Mesoporous Silica Films

**Figure 1:**
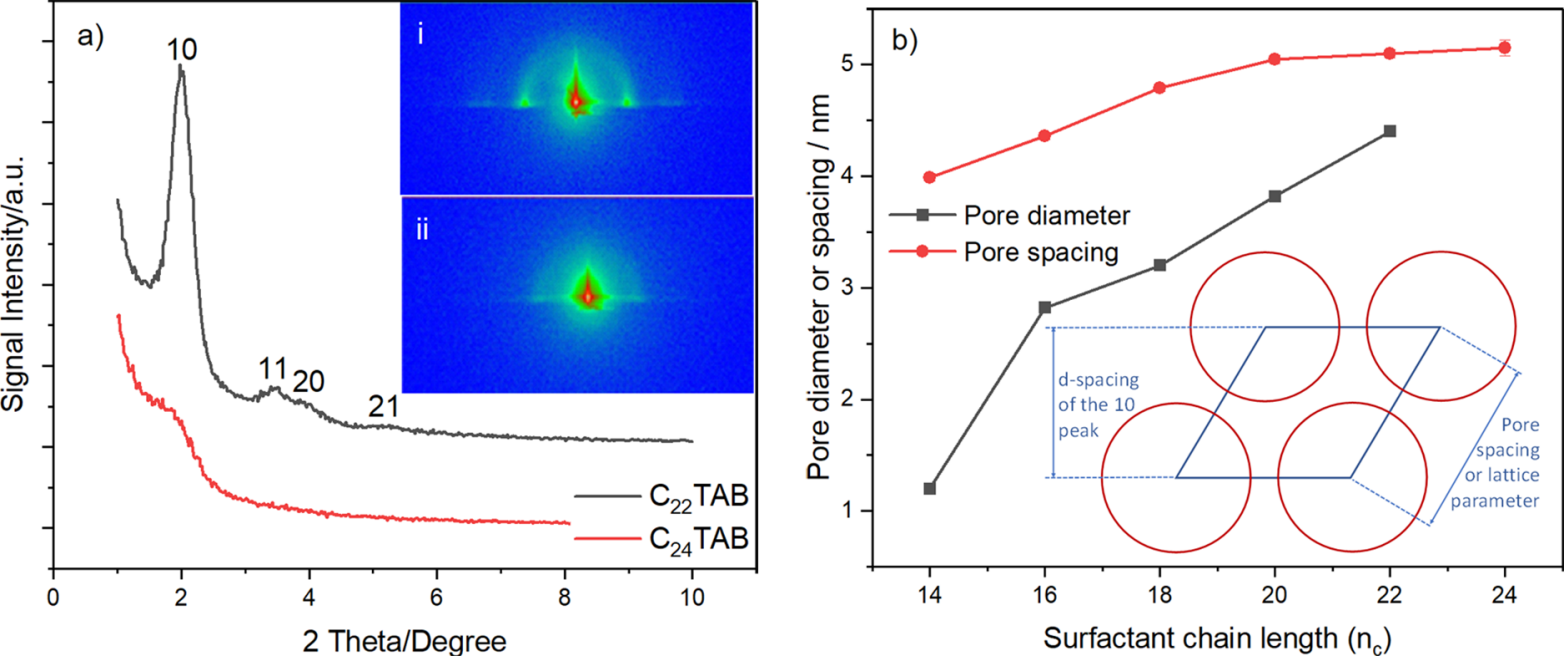
(a) 1D in-plane GISAXS patterns of EASA films with C_22_TAB and C_24_TAB deposited at a potential of −1.25 V (vs Ag/Ag^+^) for 20 s on ITO electrodes [inset: 2D GISAXS patterns for (i) C_22_TAB- and (ii) C_24_TAB-templated EASA films] and a (b) plot of pore spacing (nm) of the 10 reflection and pore diameter (nm) determined using EP as a function of surfactant chain lengths of EASA films grown with C_14_TAB,^32^ C_16_TAB, C_18_TAB, C_20_TAB, C_22_TAB, and C_24_TAB. Furthermore, the GISAXS data are given in the Supporting Information (Figures S10–S13).

The pore ordering and alignment in mesoporous silica were determined by GISAXS measurements. The 1D in-plane scattering pattern for the EASA film grown with C_22_TAB is shown in Figure 1, and contains the typical peaks for mesoporous materials with a hexagonal arrangement of cylindrical pores in *P*6*mm* symmetry, namely the SBA-15^34,35^ and MCM^36,37^ families of materials. The incident beam angle found to provide the most intense diffraction features was 0.25°, and the 10, 11, 20, and 21 peaks were observed at 2.00, 3.43, 3.96, and 5.22°, respectively. As calculated from eqs 1 and 2, the 10 peak position corresponds to a *d*-spacing of 4.42 nm and the resulting pore spacing/lattice parameter, *a*_H_ is 5.10 nm for the silica film with C_22_TAB. This set of peaks was observed for films produced with the surfactants from C_14_–C_22_.
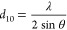
1
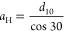
2

In contrast, the addition of C_24_TAB to the sol electrolyte resulted in a disordered sol–gel film, as indicated by the 1D in-plane GISAXS data shown in Figure 1. A clear reduction in the intensity of the broad 10 diffraction peak and a total loss of the 11 and 20 diffraction peaks were observed, indicating a significant loss of the hexagonal order. C_22_TAB is the maximum chain length with which we could obtain ordered porosity, and thus this approach allows an expansion in the pore spacing of ordered films up to 5.10 nm.

The 2D GISAXS image of the EASA film templated with C_22_TAB is presented in the insets to Figure 1i. The four in-plane spots in the horizontal plane relate to the 10, 11, 20, and 21 reflections discussed in the previous paragraph. The position of these spots in the horizontal plane shows that the pores are oriented vertically, that is, perpendicular to the plane of the substrate. The out-of-plane rings of low intensity are also observed at similar *d*-spacings to the in-plane spots, and these features increase in intensity with longer deposition times. Goux et al.^31^ reported similar GISAXS features for C_16_TAB-generated EASA films due to the growth of silica spheres at the surface with random alignment of the hexagonal pores. The second inset to Figure 1ii illustrates the 2D GISAXS data obtained for the EASA film templated with C_24_TAB after surfactant removal. The position of the 10 diffraction spots in the horizontal plane shows this film also has vertically aligned pores, but the hexagonal domains have very little longer-range order, which also accounts for the low intensity of the 1D GISAXS pattern.

The pore spacings as a function of the surfactant chain length (C_14,_ C_16_, C_18_, C_20_, C_22_, and C_24_) for a range of EASA films are also shown in Figure 1. An increase in the pore spacing values upon increasing the surfactant size is observed. This indicates a likely expansion of the pores, but does not account for the thickness of the pore walls and hence is not a direct measure of changing the pore diameter. The pore spacing values start to plateau at longer chain lengths (C_22_ and C_24_), suggesting that the effect on the pore diameter is reaching a limit using these linear chain surfactants.

**Figure 2:**
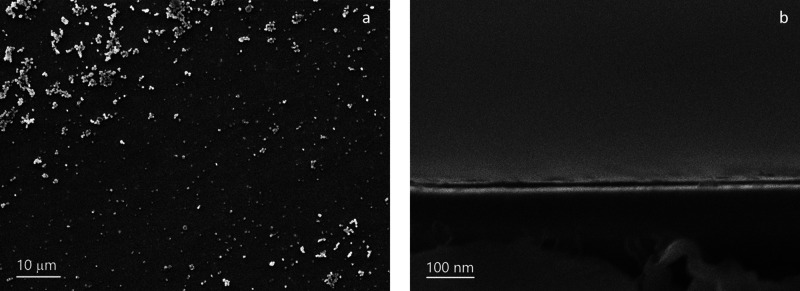
(a) Top view and (b) cross-sectional SEM image of a C_18_TAB-templated film deposited at an applied potential of −1.25 V (Ag/Ag^+^) for 20 s on an ITO electrode.

The silica films were soaked in a solution containing 0.2 mol dm^–3^ HCl in ethanol to remove the surfactant from the films. The top view SEM images (Figure 2) provided evidence that the film was free of microcracks. The coating thickness of mesoporous silica was found through cross-sectional imaging to be in the range of 100–150 nm, similar to films produced with C_16_TAB under similar conditions.^25^ The presence of silica aggregates on the electrode surface arises from the diffusion of hydroxide ions from the electrode–electrolyte interface and into the neighboring bulk solution.^38^ The diffusion layer thickness was calculated using √π*DT* (where *D* is the diffusion coefficient in which OH^–^ equals to 4 × 10^–5^ cm^2^ s^–1^ and t is the time taken for the deposition process), and is notably larger (500 μm) than the thickness of the aligned porous silica films. In this diffusion layer, silica continues to condense in the bulk solution, resulting in a layer of silica sphere aggregates on the film’s surface produced through a conventional Stöber process,^24,38^ also visible in Figure 2. This silica overgrowth can be removed using a sticky tape (Supporting Information, Figure S14), or reduced by either optimizing the sol composition, or adjusting the deposition potential and time. The “Scotch tape test” can also be used to test film adhesion on the ITO surface, and when the tape is attached directly onto the surface and peeled off the silica remains intact.

**Figure 3:**
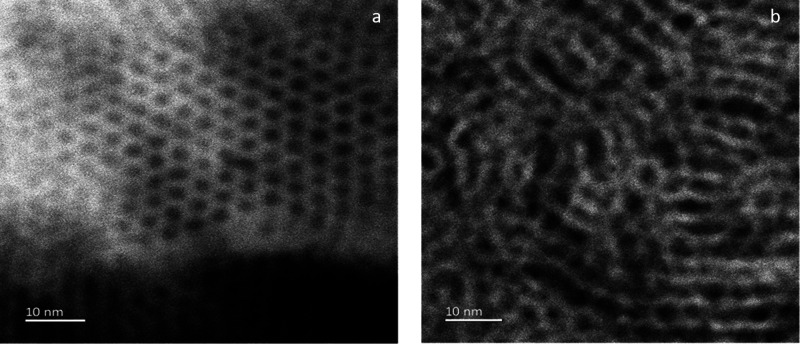
TEM images of mesoporous silica films produced with (a) C_22_TAB and (b) C_24_TAB deposited at −1.25 V (vs Ag/Ag^+^) for 20 s on ITO electrodes.

To further investigate the porous silica structure, TEM samples were prepared using EASA films prepared from C_22_TAB and C_24_TAB. The GISAXS data (Figure 1) suggested that the strong hexagonal order found with C_16_TAB could be maintained to C_22_TAB, but that with C_24_TAB experienced loss of order. The images in Figure 3 support this finding because the mesopores in the C_22_TAB-derived silica film have vertical orientation with large hexagonal domains across the film. In contrast, the TEM samples of silica films prepared with C_24_TAB retain the mesoporosity but have only very small regions of hexagonal order in the pore structure. This image was taken through a flake of silica removed from the film with a scalpel which sits flat on the TEM grid, and the pores are clearly still vertical, consistent with the GISAXS data.

### Ellipsometric Porosimetry

EP was used to examine the silica surface of EASA films grown with different sized surfactants. EP can provide detailed information about the porosity, sorption isotherms, and pore size distribution (PSD). Toluene was chosen as the probe molecule as it primarily interacts with surfaces through nonpolar dispersion forces.^39,40^ Highly polar solvents such as water are likely to produce large hysteresis because of interaction with the hydroxyl groups on the pore wall during the adsorption–desorption process.^41^ Changes in refractive index values were measured during the experiment, which allowed for the volume fraction of toluene present within the pores to be calculated using the Lorentz–Lorenz effective medium approximation.^39^

**Figure 4:**
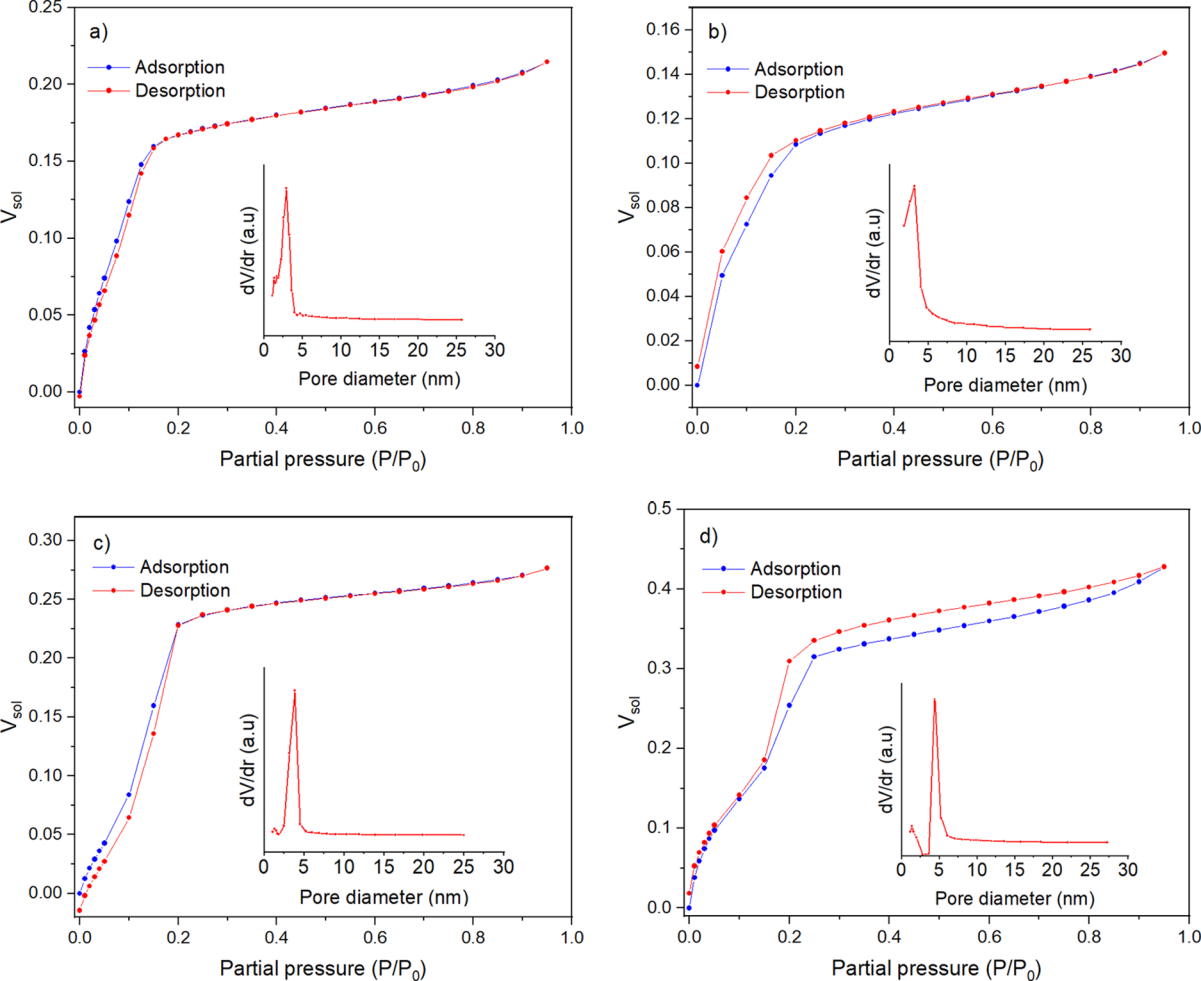
The adsorption–desorption isotherms of the EASA films grown with (a) C_16_TAB, (b) C_18_TAB, (c) C_20_TAB, and (d) C_22_TAB surfactants using toluene. The insets show the corresponding PSDs.

In Figure 4a (inset). the silica films containing C_16_TAB generated an isotherm with characteristics of Type I (b) and Type IV (b),^42^ with a very minor step noticed at around 0.09 *P*/*P*_0_ when exposed to toluene vapor. The initial increase in volume fraction up to 0.17 *P*/*P*_0_ is in line with the mixed monolayer–multilayer formation on the surface of the material and large micropore or small mesopore filling, of which the latter is most likely dominant. The isotherm, unlike typical Type I (b) and Type IV (b) shapes, does not fully plateau after the inflection point at *P*/*P*_0_ = 0.17, almost like a Type II isotherm. This indicates that some degree of heterogeneous mesoporosity is present, which is supported by the gradual decrease in the pore volume as the pore diameter increases past 4–5 nm and is also present in all the other films. The adsorption–desorption branches present minimal hysteresis, likely due to the size of the mesopores within the material being below the critical diameter for capillary condensation to occur. In comparison, nitrogen systems at −196 °C show hysteresis in pores wider than ∼4 nm,^42^ although the critical diameter for toluene vapor at ambient conditions has not been determined quantitatively.

Films made with C_18_ (Figure 4b inset) also produced an isotherm with similar characteristics to C_16_, with the step of around 0.10 *P*/*P*_0_ still observed. The curvature at which the uptake levels off is broader than in the film produced with the C_16_ surfactant, a result that can be attributed to a greater degree of heterogeneous mesoporosity for this particular sample. There is a low level of hysteresis present in the low partial pressure region of 0–0.2 *P*/*P*_0_, and the solvent volume fraction does not return to zero after desorption, likely due to some small quantity of toluene remaining trapped within the material. A common reason for this to occur is that the material contains micropores or narrow pore constrictions, in this case with a size similar to that of the kinetic diameter of toluene (0.585 nm).^43^ Micropores are common in sol–gel-derived materials due to the formation of the polymer network in the solution.^44^ No hysteresis was present at medium to high partial pressures, again suggesting that any mesopores present have a size below the critical hysteresis diameter.

The silica film grown with C_20_TAB (Figure 4c inset) exhibited similar isotherm characteristics as C_16_ and C_18_, but was found to possess a more noticeable, sharper step of around 0.10 *P*/*P*_0_, as is associated with Type IV isotherms. The sharper step implies that the transition from surface adsorption to pore filling is more distinct and points to C_20_ having a narrower PSD compared to C_16_ and C_18_, as seen in the PSD plot. This phenomenon is common in ordered mesoporous silicas, with a study by Calleja et al.^45^ observing a similar isotherm shape for MCM-41 exposed to nitrogen, although the transition from the surface to pore filling was observed to occur at a partial pressure of between 0.15 and 0.2 *P*/*P*_0_. There was also no hysteresis observed at higher partial pressures in the isotherm.

For the film with C_22_TAB, the shape of the isotherm matches closest to that of a Type IV (a), which is common for mesoporous materials. The step in the isotherm has shifted toward a higher partial pressure of around 0.15 *P*/*P*_0_, although still lower than that of typical Type IV (a) isotherms. It also presents hysteresis, which suggests the critical mesopore diameter has been reached and capillary condensation occurs. Both these observations are consequences of an increase in the pore size. The hysteresis loop most closely matches the H4 and H5 types, which have been associated with micro–mesoporous carbons and templated silicas, respectively.^42^

Figure 4 also shows the PSD curves for the silica films with increasing chain lengths, C_16_–C_22_, which were calculated according to the modified Kelvin equation where the pores are assumed to have cylindrical symmetry.^34^ These plots display peak pore sizes in the region of 2.82, 3.24, 3.82, and 4.40 nm for the surfactants of C_16_–C_22_, respectively, which were all obtained from the desorption branch of the isotherms. The sharp peaks observed in the PSD plots are indicative of a high level of homogeneous pore size and porosity in the ordered mesoporous silica materials. Interestingly, it is also noticed that the pore diameter increases steadily upon increasing the carbon units in the surfactant chain. This compared well with the GISAXS-derived pore spacing in Figure 1, and shows a fairly consistent trend.

### Electrochemical Characterization of the Films

The pore accessibilities of the mesoporous silica films on ITO electrodes were investigated using a range of redox active probes: 0.5 mmol dm^–3^ FcMeOH, 5 mmol dm^–3^ [Ru(NH_3_)_6_]^3+/2+^, or 0.5 mmol dm^–3^ [Fe(CN)_6_]^3–/4–^ in solutions also containing 0.1 mol dm^–3^ NaNO_3_ as the supporting electrolyte. This approach has been used previously to demonstrate pore accessibility in EASA-derived silica films,^25,46^ with similar results to those shown in the Supporting Information for films made with C_18_TAB, C_20_TAB, and C_22_TAB (Figures S15–S17). The reversible redox reactions of the active species are shown in eqs 3–5. In brief, the as-prepared silica films containing surfactants have a strong suppression of the electrochemical current with the charged probes, showing the films to have a good coverage. A smaller reduction in the current and a positive shift in the oxidation/reduction potentials with ferrocene methanol are due to this probe’s solubility in the surfactant and interaction with it. After surfactant removal, the peak potential separations and the currents were similar to those observed on bare ITO with the ferrocene methanol and ruthenium hexamine probes. This suggests a high degree of pore accessibility. In the case of the hexacyanoferrate probe, somewhat lower currents and a larger peak separation with the mesoporous silica films are associated with the interaction between the negatively charged silica walls and the probe, that is, the Gibbs–Donnan effect.^46,47^
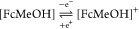
3
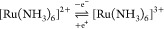
4
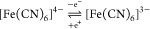
5

It is evident that extending the alkyl chain length of surfactants (C_14_, C_16_, C_18_, C_20_, and C_22_) affects the rate of diffusion of the active species into the porous silica medium.

**Figure 5:**
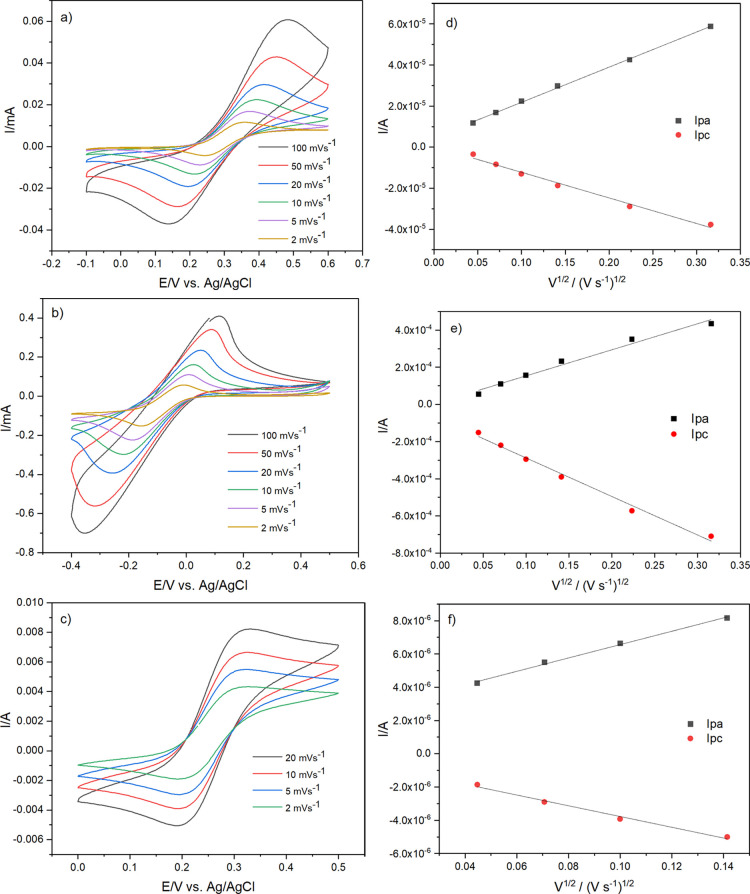
Cyclic voltammograms of (a) 0.5 mmol dm^–3^ FcMeOH, (b) 5 mmol dm^–3^ [Ru(NH_3_)_6_]^3+/2+^, and (c) 0.5 mmol dm^–3^ [Fe(CN)_6_]^4–/3–^ in 0.1 mol dm^–3^ NaNO_3_ at various scan rates {2, 5, 10, 20, 50, and 100 mV s^–1^ for the FcMeOH and [Ru(NH_3_)_6_]^3+/2+^ redox species and 2, 5, 10, and 20 mV s^–1^ for the [Fe(CN)_6_]^4–/3–^ redox couple}; the reliance of the peak current as a function of the square root of scan rates for the film containing C_22_TAB deposited at −1.25 V (vs Ag/AgCl) for 20 s on an ITO electrode. All the experiments were carried out after surfactant removal: (d) FcMeOH, (e) [Ru(NH_3_)_6_]^3+/2+^, and (f) [Fe(CN)_6_]^4–/3–^.

Figure 5 shows cyclic voltammograms recorded with a film produced from C_22_TAB (others in the Supporting Information, Figures S18–S21) at scan rates between 2 and 100 mV s^–1^ for FcMeOH and [Ru(NH_3_)_6_]^3+/2+^ and between 2 and 20 mV s^–1^ for [Fe(CN)_6_]^4–/3–^. The CV profiles became distorted at faster scan rates than this for the anionic species. The peak potential (*I*_p_) as a function of the square root of the scan rate (*v*^1/2^) is shown in Figure 5d–f. A linear trend is observed for the *I*_p_ versus *v*^1/2^ scan rate, suggesting that the current is governed mainly by diffusion.

For the electron transfer reactions of diffusional processes in the solution, it is possible to use the Randles–Sevcik (RS) equation to determine the diffusion coefficients of redox active species through the porous silica medium. The RS equation (eq 6) describes how the current increases exponentially with the square root of the scan rate. *I*_p_ is the peak current, *n* is the number of electrons transferred in the oxidation and reduction reactions, *A* is the electrode area, *D* is the diffusion coefficient, *T* is the temperature, and *C* is the concentration of the bulk solution. In the CV plots, the peak potential separations are greater than 59 mV for the various redox active species, indicating effects due to *iR* drop and possible quasi-reversible behavior. The RS equation applies to electrochemically reversible systems, so the calculated diffusion coefficients will be affected by resistance effects. The shift in the peak current position with the scan rate is likely due to the *iR* drop. In Figure 5b, the increase in the concentration of the ruthenium species results in a dramatic increase in the current in comparison to the lower concentrations of FcMeOH and [Fe(CN)_6_]^4–/3–^ species, indicating a greater *iR* drop.
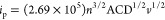
6

The RS equation may be modified to take account of the proportion of the surface that is accessible to the electrolyte by including a porosity parameter as shown in eq 7.^48^ Porosity (φ) values calculated from the pore spacing and pore diameter measurements were in the range between 0.1 and 0.7 (Supporting Information, Table S1). The apparent diffusion coefficients calculated from the gradient of the linear plots, and assuming that the concentrations of the redox species in the film are the same as those in the bulk solution, are given in Table 1 together with the surfactants and the active species. The apparent diffusion coefficient increases with the size of the surfactant used to produce the film, providing further evidence of pore expansion and easier electrochemical access through the pores with the surfactant chain length. However, it is noted that the silica film will change the concentration of the active species in the pores relative to the bulk solution to some extent. For example, the *D* values for [Fe(CN)_6_]^4–/3–^ species are smaller than for the FcMeOH and [Ru(NH_3_)_6_]^3+/2+^ species due to the Gibbs–Donnan effect mentioned earlier.
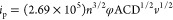
7

**Table 1:** Apparent Diffusion Coefficients of EASA Films with Surfactants of Increasing Chain Length Using a Range of Redox Active Probes

Surfactant	*D*_[FcMeOH]_ (cm^2^ s^–1^)	*D*_[Ru(NH_3_)_6_]^3+/2+^_ (cm^2^ s^–1^)	*D*_[Fe(CN)_6_]^4–/3–^_ (cm^2^ s^–1^)
C_14_TAB	7.4 × 10–7	6.8 × 10–7	1.4 × 10–7
C_16_TAB	8.9 × 10–7	8.2 × 10–7	2.8 × 10–7
C_18_TAB	9.4 × 10–7	1.1 × 10–6	3.8 × 10–7
C_20_TAB	2.3 × 10–6	2.2 × 10–6	5.2 × 10–7
C_22_TAB	4.6 × 10–6	3.5 × 10–6	8.6 × 10–7

## Conclusions

Ordered mesoporous silica films with vertically aligned pores were produced by an electrochemically driven sol–gel method (EASA). The pore size was increased by extending the alkyl chain length in the [Me_3_NR]Br surfactant from 16 to 22 carbons using quaternary ammonium salts, which resulted in larger pore diameters together with retained order. The film became disordered when adding C_24_TAB into the sol electrolyte, indicating a constraint in response to micelle expansion using linear chained surfactants, although the vertical pore orientation was retained. The ion diffusion rates using redox active probes were found to increase with the surfactant size and hence the pore size. The pore size increased from 2.8 nm using C_16_TAB to 4.4 nm using C_22_TAB. The expanded pores in these vertically aligned mesoporous silica films may make them more amenable to use as templates for the electrochemical deposition of nanowires, and change their behavior in sensing applications.

## Supporting Information Available

NMR and mass spectra; further GISAXS data; cyclic voltammograms and diffusion coefficient calculations; and tabulated pore spacing and porosity data (PDF)
