# Supplementary material for: Increasing the Diameter of Vertically Aligned, Hexagonally Ordered Pores in Mesoporous Silica Thin Films
Source: Langmuir. 2022 Feb 8;38(7):2257–66. doi: 10.1021/acs.langmuir.1c02854 (PMC9097518; doi:10.1021/acs.langmuir.1c02854)
Supplement: Supplementary file 1 — la1c02854_si_001.pdf [file la1c02854_si_001.pdf]

Supplementary information: Increasing the diameter of vertically aligned, hexagonally ordered pores in mesoporous silica thin films

Nabil A. N. Mohamed,[a] Yisong Han,[b] Andrew L. Hector,[a]* Anthony R. Houghton,[c] Elwin Hunter-Sellars,[c] Gillian Reid,[a] Daryl R. Williams[c] and Wenjian Zhang[a]

[a] School of Chemistry, University of Southampton, Highfield, Southampton SO17 1BJ, UK. Email A.L.Hector@soton.ac.uk

[b] Department of Physics, University of Warwick, Coventry CV4 7AL, UK

[c] Department of Chemical Engineering, Imperial College London SW7 2AZ, UK

List of supplementary figures and table

Figure S1. ^{1}H NMR spectrum of C_{20}TAB in $CDCl_3$ at 25 °C. δ/ppm 0.83 (t, CH_3, [3H]), 1.17 (br s, CH_2, [32H]), 1.26-1.37 (m, CH_2, [2H]), 1.63-1.77 (m, CH_2, [2H]), 3.41 (s, CH_3, [9H]), 3.47-3.56 (m, CH_2, [2H]).

Figure S2. ^{13}C{^{1}H} NMR spectrum of C_{20}TAB in $CDCl_3$ at 25 °C. δ/ppm 14.12, 22.70, 23.23, 26.19, 26.22, 29.23, 29.32, 29.36, 29.47, 29.54, 29.59, 29.63, 29.66, 29.68, 29.70, 29.71, 31.92, 53.41, 58.43, 67.12.

Figure S3. ^{1}H NMR spectrum of C_{22}TAB in $CDCl_3$ at 25 °C. δ/ppm 0.82 (t, CH_3, [3H]), 1.19 (br s, CH_2, [36H]), 1.29-1.37 (m, CH_2, [2H]), 1.61-1.65 (m, CH_2, [2H]), 3.41 (s, CH_3, [9H]), 3.47-3.53 (m, CH_2, [2H]).

Figure S4 ^{13}C{^{1}H} NMR spectrum of C_{22}TAB in $CDCl_3$ at 25 °C. δ/ppm 14.10, 22.72, 23.21, 26.17, 29.22, 29.27, 29.32, 29.35, 29.36, 29.46, 29.59, 29.65, 29.66, 29.69, 29.71, 31.89, 31.91, 31.93, 42.63, 53.45, 67.18, 67.21.

Figure S5. ^{1}H NMR spectrum of C_{24}TAB in $CDCl_3$ at 25 °C. δ/ppm 0.82 (t, CH_3, [3H]), 1.18 (s, CH_2, [40H]), 1.22-1.33 (m, CH_2, [42H]), 1.63-1.72 (m, CH_2, [2H]), 3.40 (s, CH_3, [9H]), 3.46-3.54 (m, CH_2, [2H]).

Figure S6. ^{13}C{^{1}H} NMR spectrum of C_{24}TAB in $CDCl_3$ at 25 °C. δ/ppm 14.12, 22.70, 23.25, 25.32, 25.33, 26.18, 29.20, 29.29, 29.33, 29.37, 29.44, 29.55, 29.58, 29.65, 29.67, 29.69, 29.71, 31.94, 43.65, 53.46, 53.51, 53.54, 53.59, 53.60.

Figure S7. Positive ion electrospray mass spectrum of the $[N(CH_3)_3(CH_2)_{19}CH_3]^+$ cation in C_{20}TAB in methanol at 25 °C (expected *m/z* = 340).

Figure S8. Positive ion electrospray mass spectrum of the $[N(CH_3)_3(CH_2)_{21}CH_3]^+$ cation in C_{22}TAB in methanol at 25 °C (expected *m/z* = 368).

Figure S9. Positive ion electrospray mass spectrum of the $[N(CH_3)_3(CH_2)_{23}CH_3]^+$ cation in C_{24}TAB in methanol at 25 °C (expected *m/z* = 396).

Figure S10. 1D in-plane GISAXS pattern of an EASA film with C_{14}TAB deposited at a potential of -1.25 V (vs. Ag/Ag^+) for 20 s on an ITO electrode. Inset: 2D in-plane GISAXS pattern of C_{14}TAB templated EASA film.

Figure S11. 1D in-plane GISAXS pattern of an EASA film with C_{16}TAB deposited at a potential of -1.25 V (vs. Ag/Ag^+) for 20 s on an ITO electrode. Inset: 2D in-plane GISAXS pattern of C_{16}TAB templated EASA film.

Figure S12. 1D in-plane GISAXS pattern of an EASA film with C_{18}TAB deposited at a potential of -1.25 V (vs. Ag/Ag^+) for 20 s on an ITO electrode. Inset: 2D in-plane GISAXS pattern of C_{18}TAB templated EASA film.

Figure S13. 1D in-plane GISAXS pattern of an EASA film with C_{20}TAB deposited at a potential of -1.25 V (vs. Ag/Ag^+) for 20 s on an ITO electrode. Inset: 2D in-plane GISAXS pattern of C_{20}TAB templated EASA film.

Figure S14. A photograph of a mesoporous silica film produced by C_{20}TAB deposited at a potential of -1.25 V (vs. Ag/Ag^+) for 20 s on an ITO electrode.

Figure S15. Cyclic Voltammograms (20 mV s^{-1} sweep rate) of (a) 0.5 mmol dm^{-3} [FcMeOH], (b) 5 mmol dm^{-3} $[Ru(NH_3)_6]^{3+/2+}$ and (c), (d) 0.5 mmol dm^{-3} $[Fe(CN)_6]^{3-/4-}$ in 0.1 mol dm^{-3} $NaNO_{3(aq)}$ on bare ITO electrode (black line), and with a silica film before surfactant removal (red line) and after surfactant removal (blue line). The generated mesoporous silica film using C_{18}TAB as the surfactant was deposited at -1.25 V (vs. Ag/AgCl) for 20 s on an ITO electrode.

Figure S16. Cyclic Voltammograms (20 mV s^{-1} sweep rate) of (a) 0.5 mmol dm^{-3} [FcMeOH], (b) 5 mmol dm^{-3} $[Ru(NH_3)_6]^{3+/2+}$ and (c) 0.5 mmol dm^{-3} $[Fe(CN)_6]^{3-/4-}$ in 0.1 mol dm^{-3} $NaNO_3$(aq) on bare ITO electrode (black line), before surfactant removal (red line) and after surfactant removal (blue line). The generated mesoporous silica film using C_{20}TAB as the surfactant was deposited at -1.25 V (vs. Ag/AgCl) for 20 s on an ITO electrode.

Figure S17. Cyclic Voltammograms (20 mV s^{-1} sweep rate) of (a) 0.5 mmol dm^{-3} [FcMeOH], (b) 5 mmol dm^{-3} $[Ru(NH_3)_6]^{3+/2+}$ and (c) 0.5 mmol dm^{-3} $[Fe(CN)_6]^{3-/4-}$ in 0.1 mol dm^{-3} $NaNO_3$(aq) on bare ITO electrode (black line), before surfactant removal (red line) and after surfactant removal (blue line). The generated mesoporous silica film using C_{22}TAB as the surfactant was deposited at -1.25 V (vs. Ag/AgCl) for 20 s on an ITO electrode.

Figure S18. The CVs of (a) 0.5 mmol dm^{-3} [FcMeOH], (b) 5 mmol dm^{-3} $[Ru(NH_3)_6]^{3+/2+}$ and (c) 0.5 mmol dm^{-3} $[Fe(CN)_6]^{4-/3-}$ at various scan rates (2, 5, 10, 20, 50 and 100 mV s^{-1} for [FcMeOH] and $[Ru(NH_3)_6]^{3+/2+}$ redox species and 2, 5, 10 and 20 mVs^{-1} for $[Fe(CN)_6]^{4-/3-}$ redox couple); reliance of peak current as a function of square root of scan rates for the film containing C_{14}TAB deposited at -

1.25 V (vs. Ag/AgCl) for 20 s on an ITO electrode. All experiments were carried out after surfactant removal: (d) [FcMeOH], (e) $[Ru(NH_3)_6]^{3+/2+}$ and (f) $[Fe(CN)_6]^{4-/3-}$.

Figure S19. The CV's of (a) 0.5 mmol dm^{-3} [FcMeOH], (b) 5 mmol dm^{-3} $[Ru(NH_3)_6]^{3+/2+}$ and (c) 0.5 mmol dm^{-3} $[Fe(CN)_6]^{4-/3-}$ at various scan rates (2, 5, 10, 20, 50 and 100 mV s^{-1} for [FcMeOH] and $[Ru(NH_3)_6]^{3+/2+}$ redox species and 2, 5, 10 and 20 mVs^{-1} for $[Fe(CN)_6]^{4-/3-}$ redox couple); reliance of peak current as a function of square root of scan rates for the film containing $C_{16}TAB$ deposited at -1.25 V (vs. Ag/AgCl) for 20 s on an ITO electrode. All experiments were carried out after surfactant removal: (d) [FcMeOH], (e) $[Ru(NH_3)_6]^{3+/2+}$ and (f) $[Fe(CN)_6]^{4-/3-}$.

Figure S20. The CV's of (a) 0.5 mmol dm^{-3} [FcMeOH], (b) 5 mmol dm^{-3} $[Ru(NH_3)_6]^{3+/2+}$ and (c) 0.5 mmol dm^{-3} $[Fe(CN)_6]^{4-/3-}$ at various scan rates (2, 5, 10, 20, 50 and 100 mV s^{-1} for [FcMeOH] and $[Ru(NH_3)_6]^{3+/2+}$ redox species and 2, 5, 10 and 20 mVs^{-1} for $[Fe(CN)_6]^{4-/3-}$ redox couple); reliance of peak current as a function of square root of scan rates for the film containing $C_{18}TAB$ deposited at -1.25 V (vs. Ag/AgCl) for 20 s on an ITO electrode. All experiments were carried out after surfactant removal: (d) [FcMeOH], (e) $[Ru(NH_3)_6]^{3+/2+}$ and (f) $[Fe(CN)_6]^{4-/3-}$.

Figure S21. The CV's of (a) 0.5 mmol dm^{-3} [FcMeOH], (b) 5 mmol dm^{-3} $[Ru(NH_3)_6]^{3+/2+}$ and (c) 0.5 mmol dm^{-3} $[Fe(CN)_6]^{4-/3-}$ at various scan rates (2, 5, 10, 20, 50 and 100 mV s^{-1} for [FcMeOH] and $[Ru(NH_3)_6]^{3+/2+}$ redox species and 2, 5, 10 and 20 mVs^{-1} for $[Fe(CN)_6]^{4-/3-}$ redox couple); reliance of peak current as a function of square root of scan rates for the film containing $C_{20}TAB$ deposited at -1.25 V (vs. Ag/AgCl) for 20 s on an ITO electrode. All experiments were carried out after surfactant removal: (d) [FcMeOH], (e) $[Ru(NH_3)_6]^{3+/2+}$ and (f) $[Fe(CN)_6]^{4-/3-}$.

Table S1. The porosity values (φ) determined from the pore diameter and pore spacing measurements.

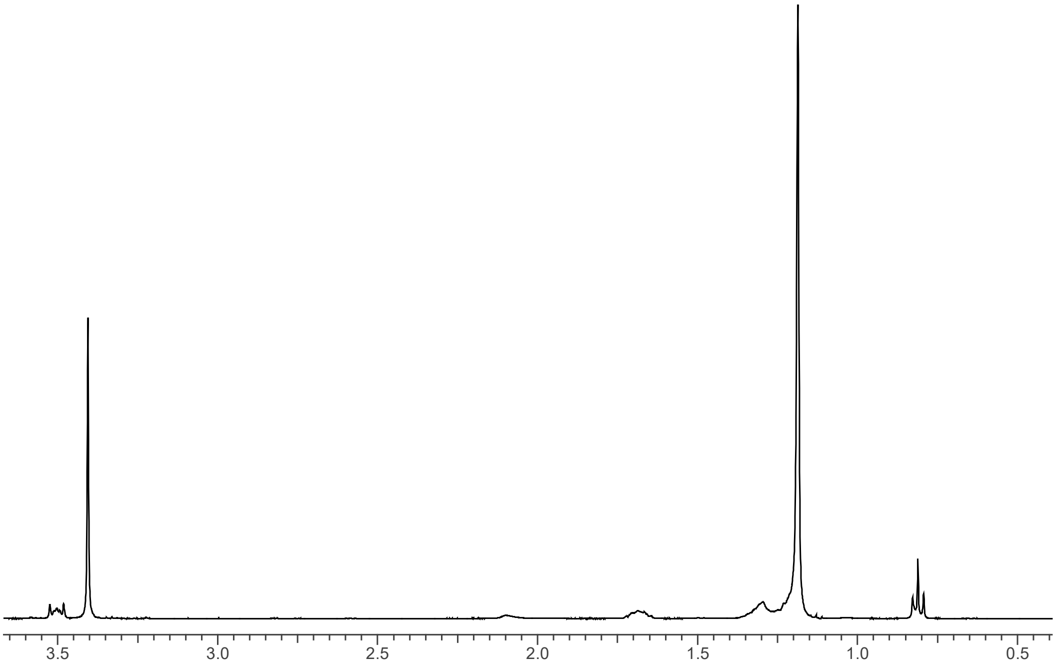

Figure S1. ^{1}H NMR spectrum of C_{20}TAB in $CDCl_3$ at 25 °C. δ/ppm 0.83 (t, CH_3, [3H]), 1.17 (br s, CH_2, [32H]), 1.26-1.37 (m, CH_2, [2H]), 1.63-1.77 (m, CH_2, [2H]), 3.41 (s, CH_3, [9H]), 3.47-3.56 (m, CH_2, [2H]).

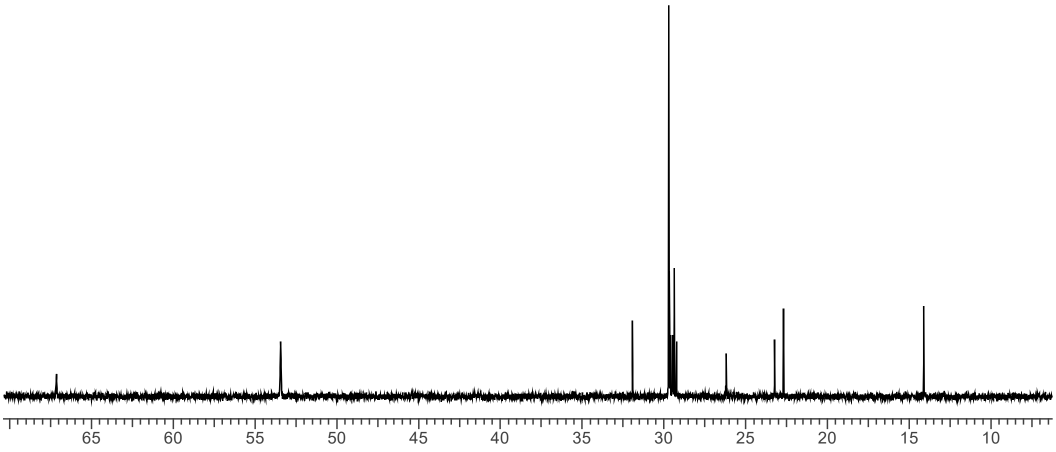

Figure S2. ^{13}C{^{1}H} NMR spectrum of C_{20}TAB in $CDCl_3$ at 25 ℃. δ/ppm 14.12, 22.70, 23.23, 26.19, 26.22, 29.23, 29.32, 29.36, 29.47, 29.54, 29.59, 29.63, 29.66, 29.68, 29.70, 29.71, 31.92, 53.41, 58.43, 67.12.

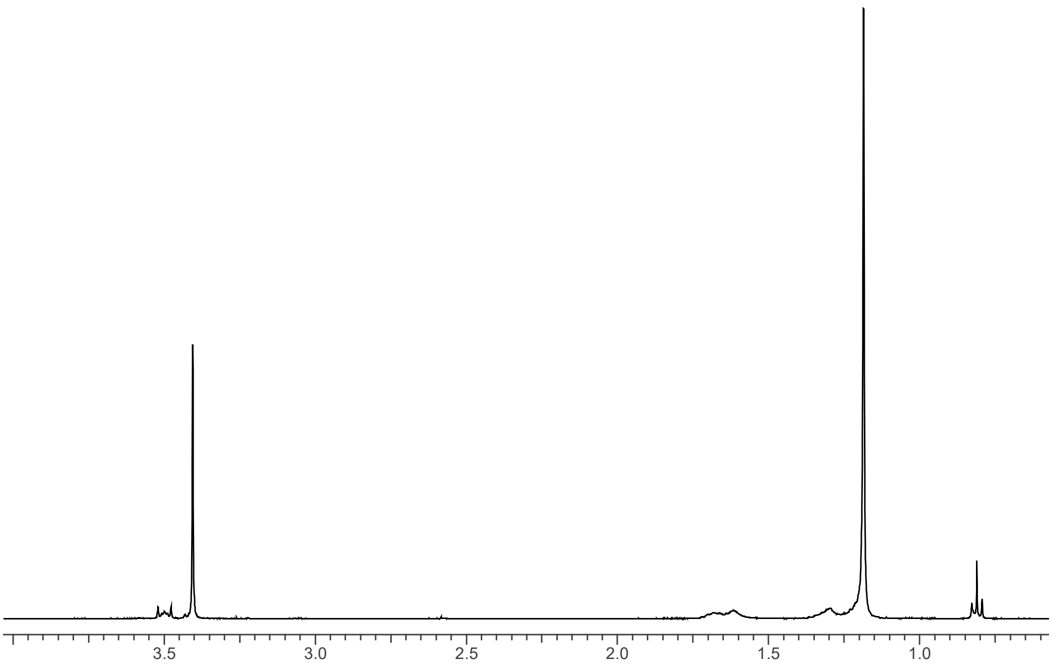

Figure S3. ^{1}H NMR spectrum of C_{22}TAB in $CDCl_3$ at 25 °C. δ/ppm 0.82 (t, CH_3, [3H]), 1.19 (br s, CH_2, [36H]), 1.29-1.37 (m, CH_2, [2H]), 1.61-1.65 (m, CH_2, [2H]), 3.41 (s, CH_3, [9H]), 3.47-3.53 (m, CH_2, [2H]).

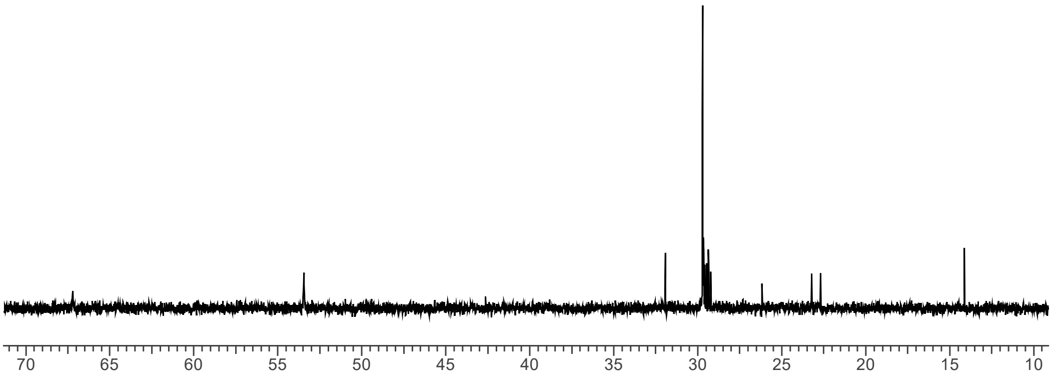

Figure S4. ^{13}C{^{1}H} NMR spectrum of C_{22}TAB in $CDCl_3$ at 25 ℃. δ/ppm 14.10, 22.72, 23.21, 26.17, 29.22, 29.27, 29.32, 29.35, 29.36, 29.46, 29.59, 29.65, 29.66, 29.69, 29.71, 31.89, 31.91, 31.93, 42.63, 53.45, 67.18, 67.21.

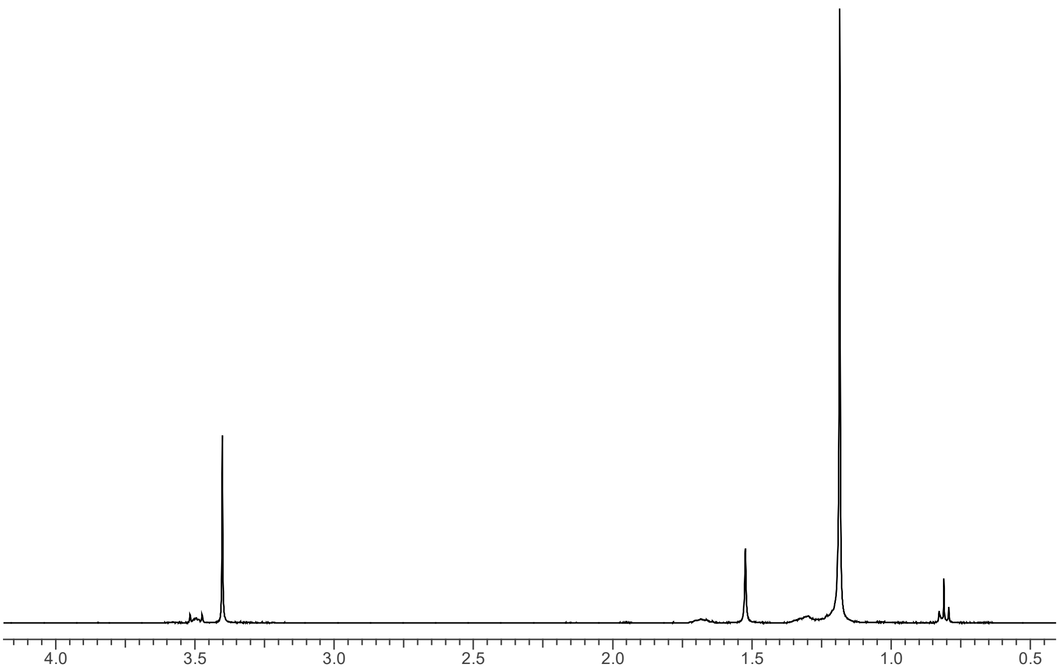

Figure S5. ^{1}H NMR spectrum of $C_{24}TAB$ in $CDCl_3$ at 25 °C. δ/ppm 0.82 (t, CH_3, [3H]), 1.18 (s, CH_2, [40H]), 1.22-1.33 (m, CH_2, [42H]), 1.63-1.72 (m, CH_2, [2H]), 3.40 (s, CH_3, [9H]), 3.46-3.54 (m, CH_2, [2H]).

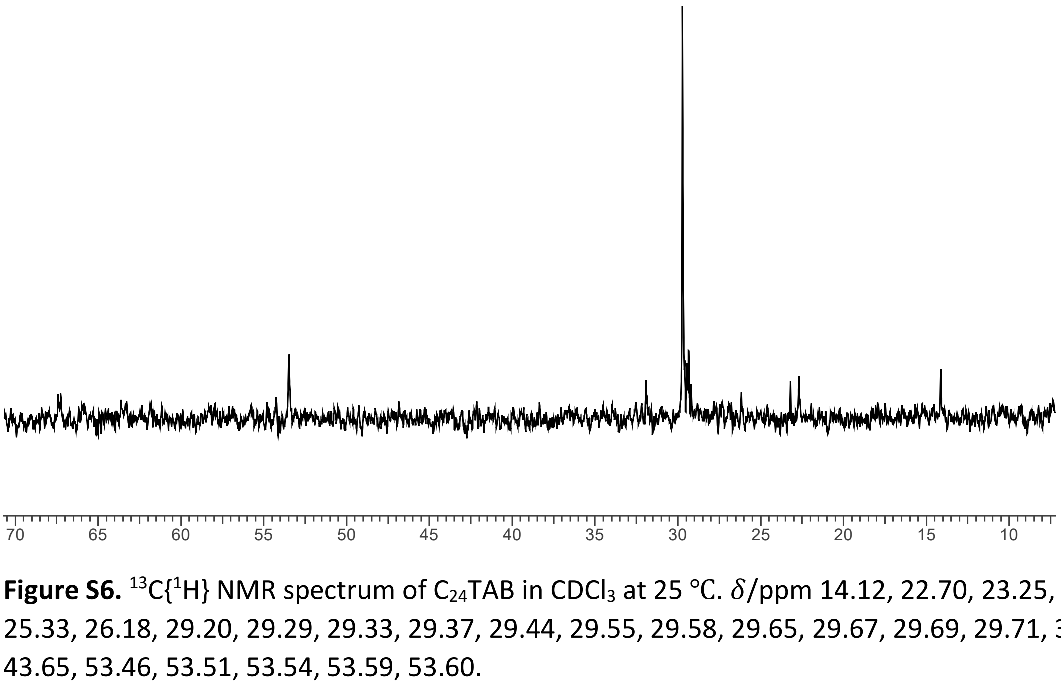

Figure S6. $^{13}C\{^{1}H\}$ NMR spectrum of $C_{24}TAB$ in $CDCl_3$ at 25 °C. δ/ppm 14.12, 22.70, 23.25, 25.32, 25.33, 26.18, 29.20, 29.29, 29.33, 29.37, 29.44, 29.55, 29.58, 29.65, 29.67, 29.69, 29.71, 31.94, 43.65, 53.46, 53.51, 53.54, 53.59, 53.60.

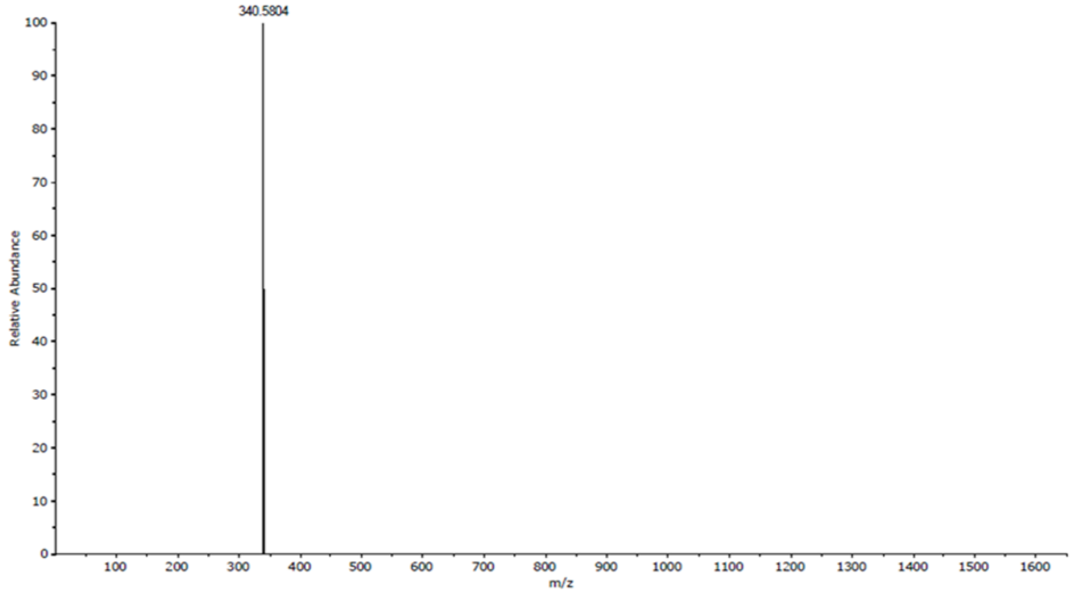

Figure S7. Positive ion electrospray mass spectrum of the $[N(CH_3)_3(CH_2)_{19}CH_3]^+$ cation in $C_{20}TAB$ in methanol at 25 °C (expected *m/z* = 340).

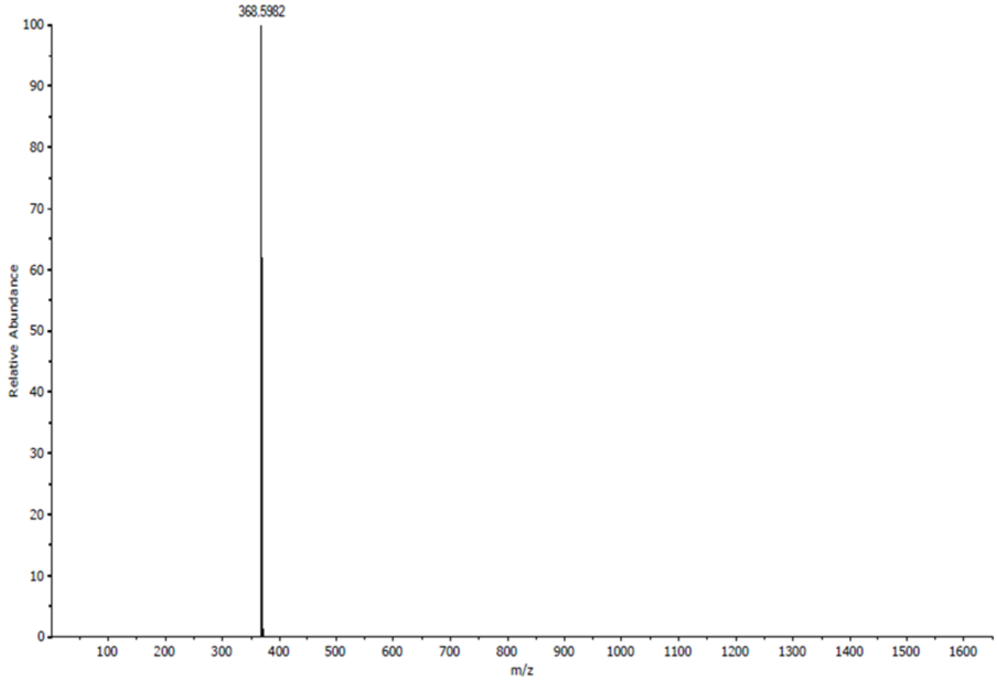

Figure S8. Positive ion electrospray mass spectrum of the $[N(CH_3)_3(CH_2)_{21}CH_3]^+$ cation in $C_{22}TAB$ in methanol at 25 °C (expected *m/z* = 368).

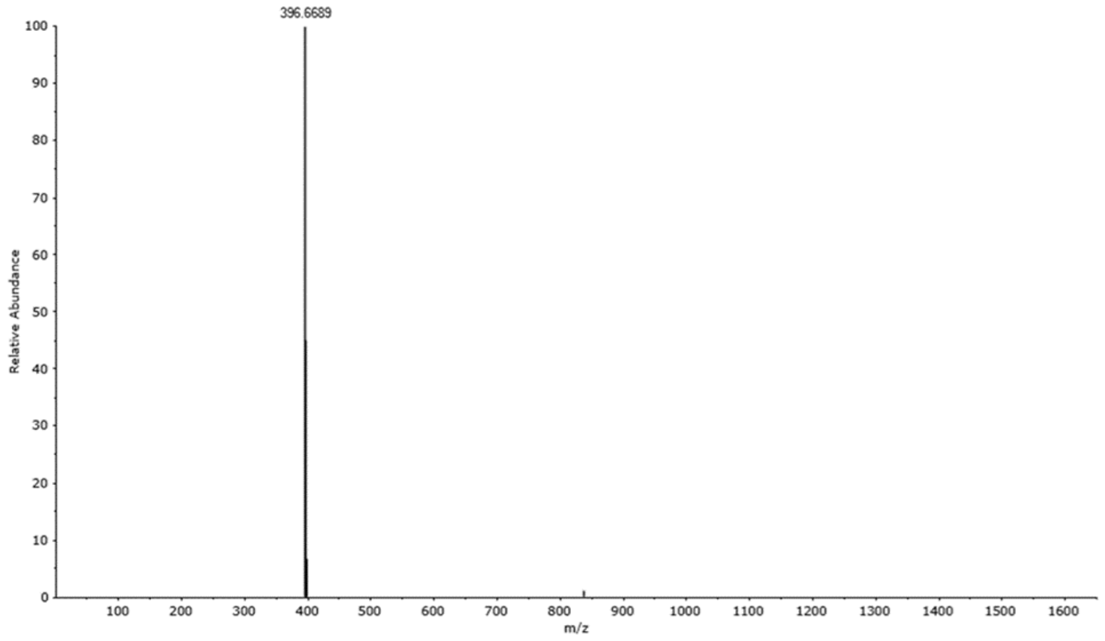

Figure S9. Positive ion electrospray mass spectrum of the $[N(CH_3)_3(CH_2)_{23}CH_3]^+$ cation in $C_{24}TAB$ in methanol at 25 °C (expected *m/z* = 396).

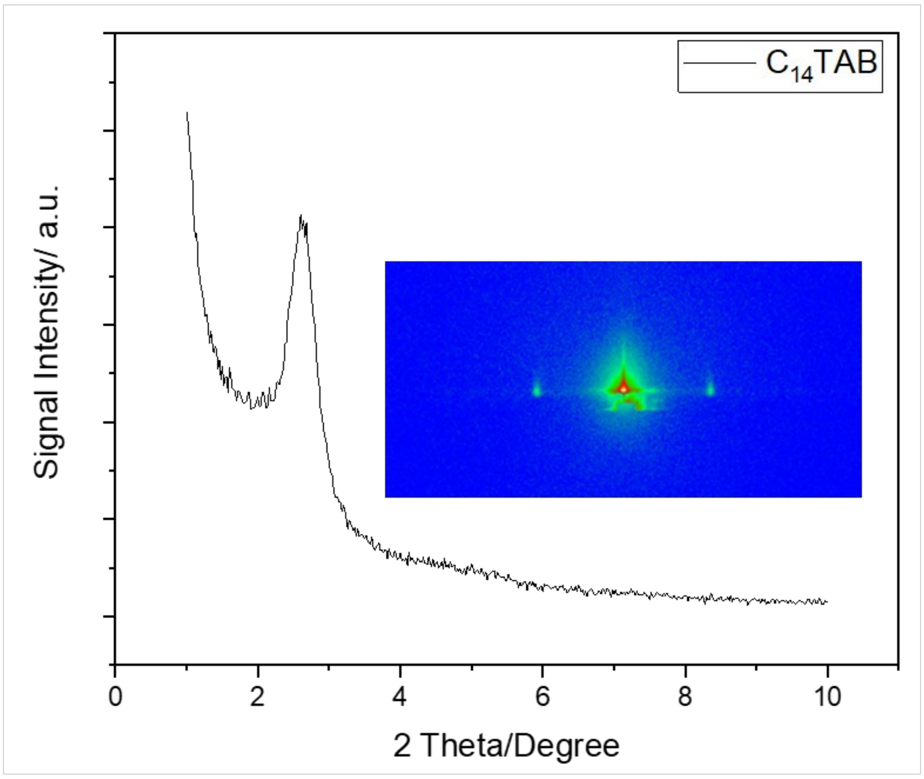

Figure S10. 1D in-plane GISAXS pattern of an EASA film with $C_{14}TAB$ deposited at a potential of -1.25 V (vs. Ag/Ag^+) for 20 s on an ITO electrode. Inset: 2D in-plane GISAXS pattern of $C_{14}TAB$ templated EASA film.

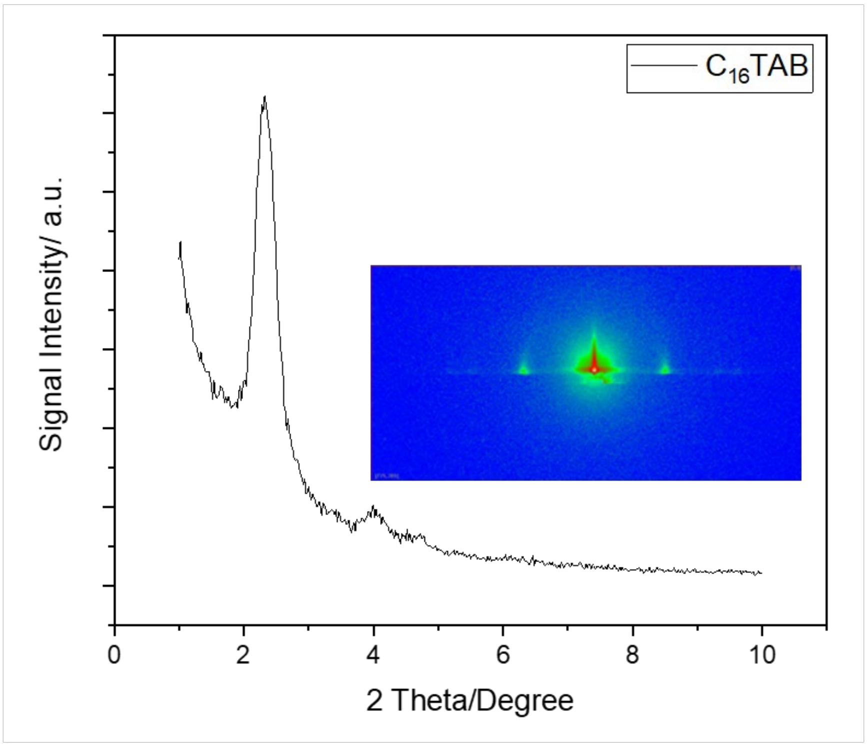

Figure S11. 1D in-plane GISAXS pattern of an EASA film with $C_{16}TAB$ deposited at a potential of -1.25 V (vs. Ag/Ag^+) for 20 s on an ITO electrode. Inset: 2D in-plane GISAXS pattern of $C_{16}TAB$ templated EASA film.

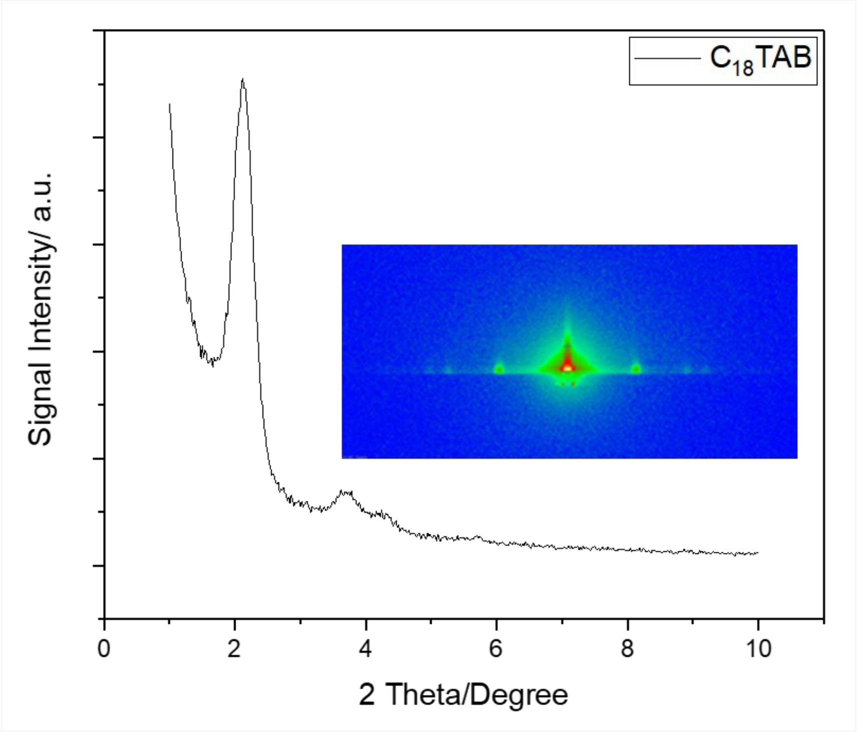

Figure S12. 1D in-plane GISAXS pattern of an EASA film with $C_{18}TAB$ deposited at a potential of -1.25 V (vs. Ag/Ag^+) for 20 s on an ITO electrode. Inset: 2D in-plane GISAXS pattern of $C_{18}TAB$ templated EASA film.

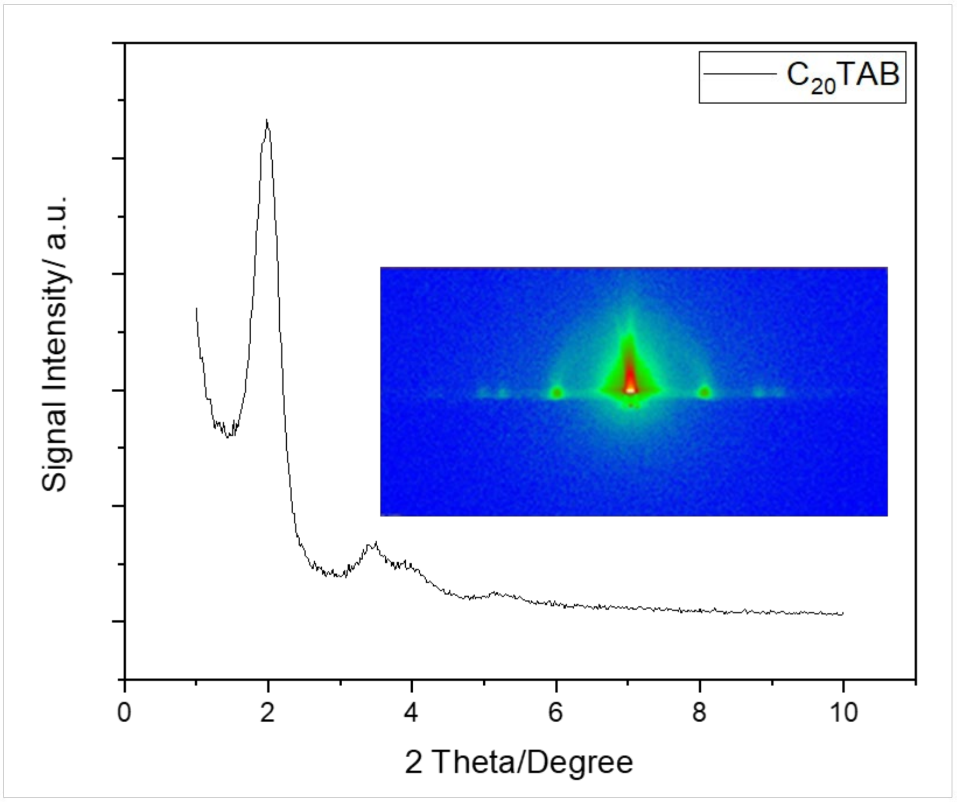

Figure S13. 1D in-plane GISAXS pattern of an EASA film with $C_{20}TAB$ deposited at a potential of -1.25 V (vs. Ag/Ag$^+$) for 20 s on an ITO electrode. Inset: 2D in-plane GISAXS pattern of $C_{20}TAB$ templated EASA film.

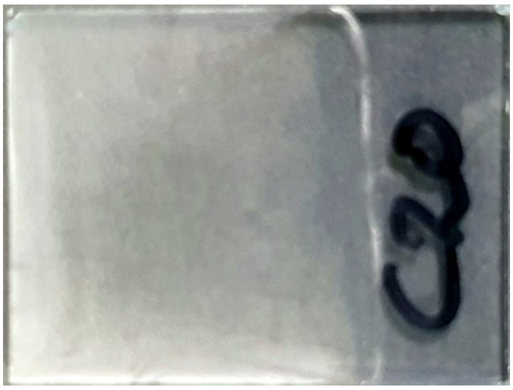

Figure S14. A photograph of a mesoporous silica film produced by $C_{20}TAB$ deposited at a potential of -1.25 V (vs. Ag/Ag$^+$) for 20 s on an ITO electrode.

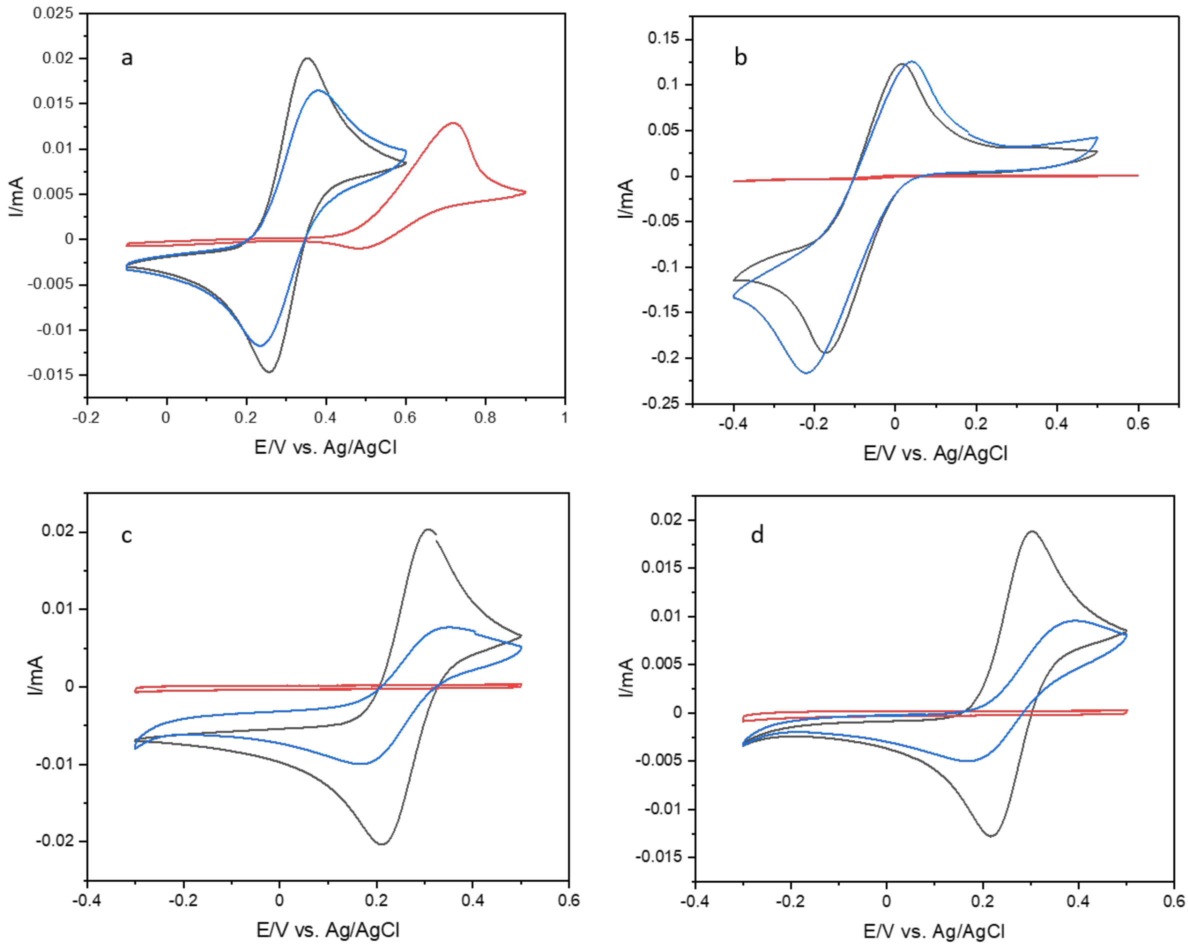

Figure S15. Cyclic Voltammograms (20 mV s^{-1} sweep rate) of (a) 0.5 mmol dm^{-3} [FcMeOH], (b) 5 mmol dm^{-3} $[Ru(NH_3)_6]^{3+/2+}$ and (c), (d) 0.5 mmol dm^{-3} $[Fe(CN)_6]^{3-/4-}$ in 0.1 mol dm^{-3} $NaNO_{3(aq)}$ on bare ITO electrode (black line), and with a silica film before surfactant removal (red line) and after surfactant removal (blue line). The generated mesoporous silica film using C_{18}TAB as the surfactant was deposited at -1.25 V (vs. Ag/AgCl) for 20 s on an ITO electrode.

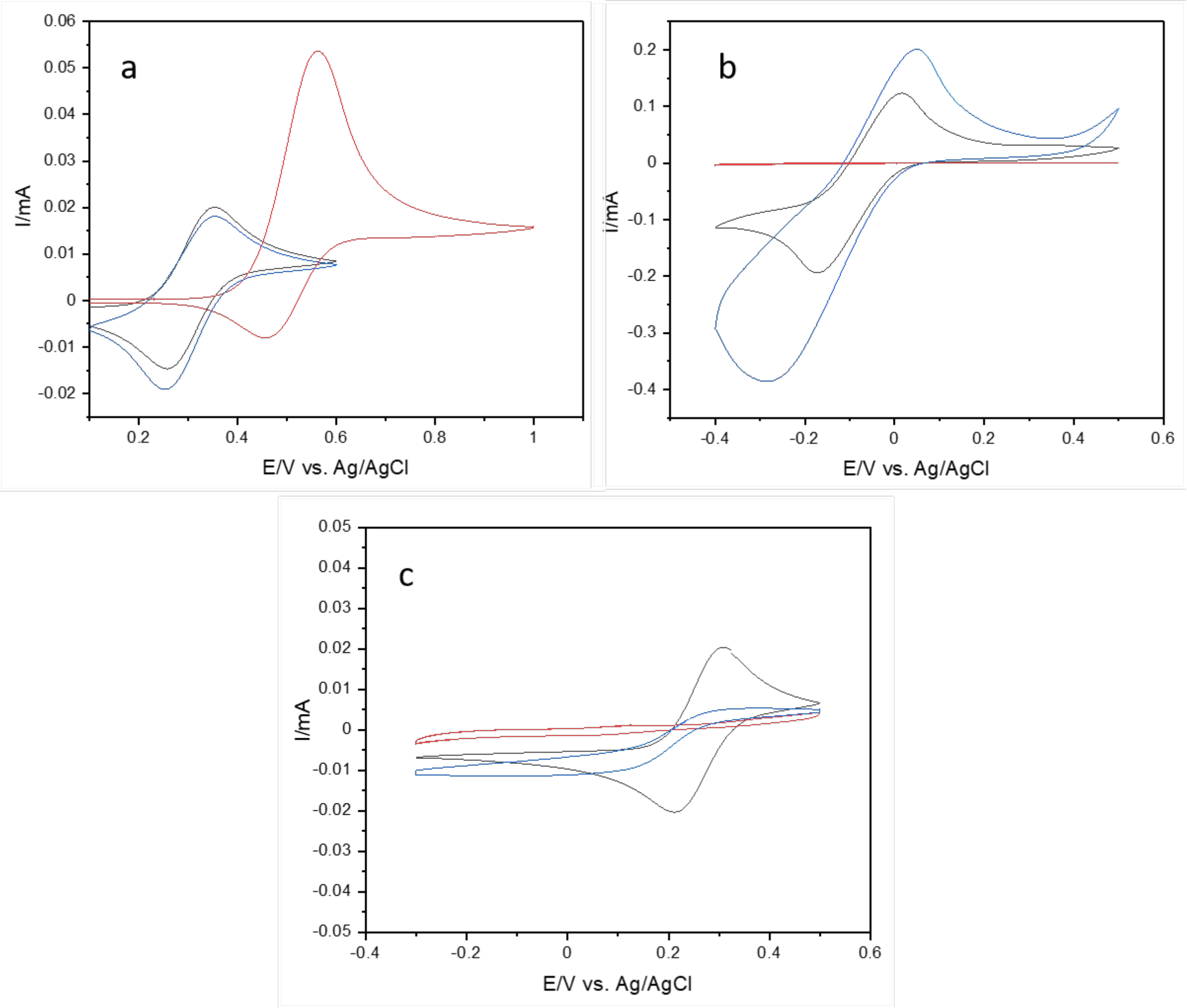

Figure S16. Cyclic Voltammograms (20 mV s^{-1} sweep rate) of (a) 0.5 mmol dm^{-3} [FcMeOH], (b) 5 mmol dm^{-3} $[Ru(NH_3)_6]^{3+/2+}$ and (c) 0.5 mmol dm^{-3} $[Fe(CN)_6]^{3-/4-}$ in 0.1 mol dm^{-3} $NaNO_3$(aq) on bare ITO electrode (black line), before surfactant removal (red line) and after surfactant removal (blue line). The generated mesoporous silica film using C_{20}TAB as the surfactant was deposited at -1.25 V (vs. Ag/AgCl) for 20 s on an ITO electrode.

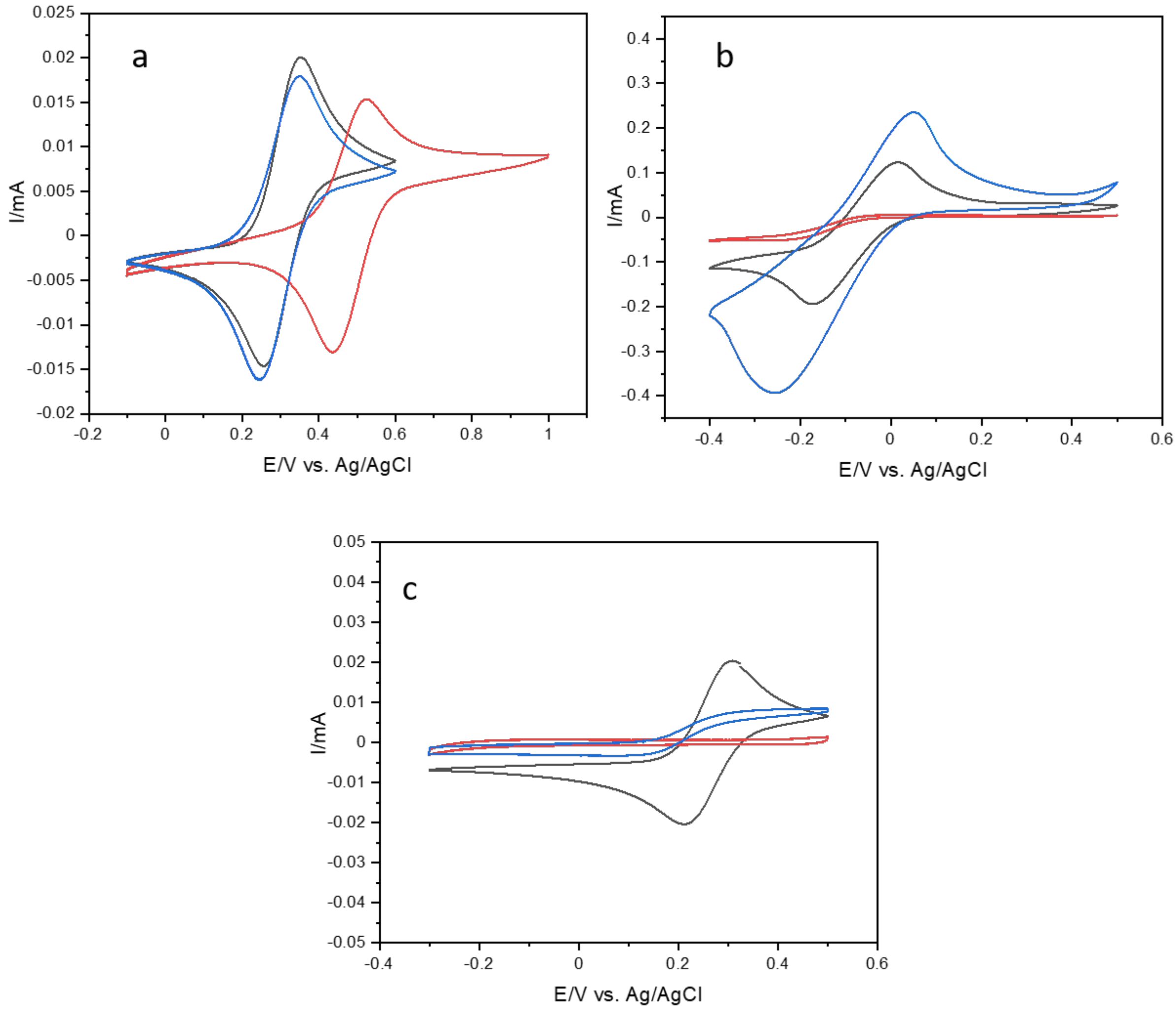

Figure S17. Cyclic Voltammograms (20 mV s^{-1} sweep rate) of (a) 0.5 mmol dm^{-3} [FcMeOH], (b) 5 mmol dm^{-3} $[Ru(NH_3)_6]^{3+/2+}$ and (c) 0.5 mmol dm^{-3} $[Fe(CN)_6]^{3-/4-}$ in 0.1 mol dm^{-3} $NaNO_3$(aq) on bare ITO electrode (black line), before surfactant removal (red line) and after surfactant removal (blue line). The generated mesoporous silica film using C_{22}TAB as the surfactant was deposited at -1.25 V (vs. Ag/AgCl) for 20 s on an ITO electrode.

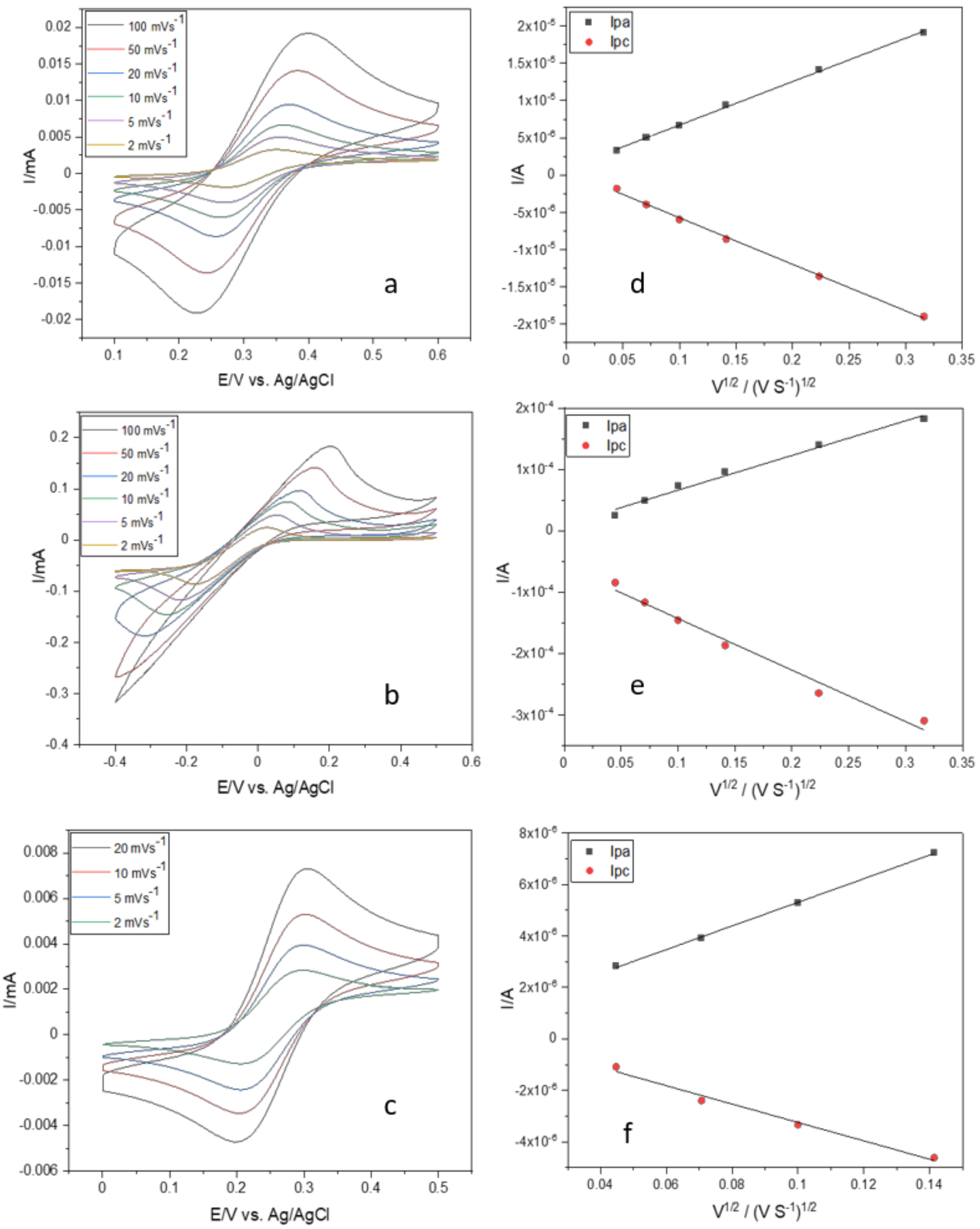

Figure S18. The CVs of (a) 0.5 mmol dm^{-3} [FcMeOH], (b) 5 mmol dm^{-3} $[Ru(NH_3)_6]^{3+/2+}$ and (c) 0.5 mmol dm^{-3} $[Fe(CN)_6]^{4-/3-}$ at various scan rates (2, 5, 10, 20, 50 and 100 mV s^{-1} for [FcMeOH] and $[Ru(NH_3)_6]^{3+/2+}$ redox species and 2, 5, 10 and 20 mVs^{-1} for $[Fe(CN)_6]^{4-/3-}$ redox couple); reliance of peak current as a function of square root of scan rates for the film containing C_{14}TAB deposited at -1.25 V (vs. Ag/AgCl) for 20 s on an ITO electrode. All experiments were carried out after surfactant removal: (d) [FcMeOH], (e) $[Ru(NH_3)_6]^{3+/2+}$ and (f) $[Fe(CN)_6]^{4-/3-}$.

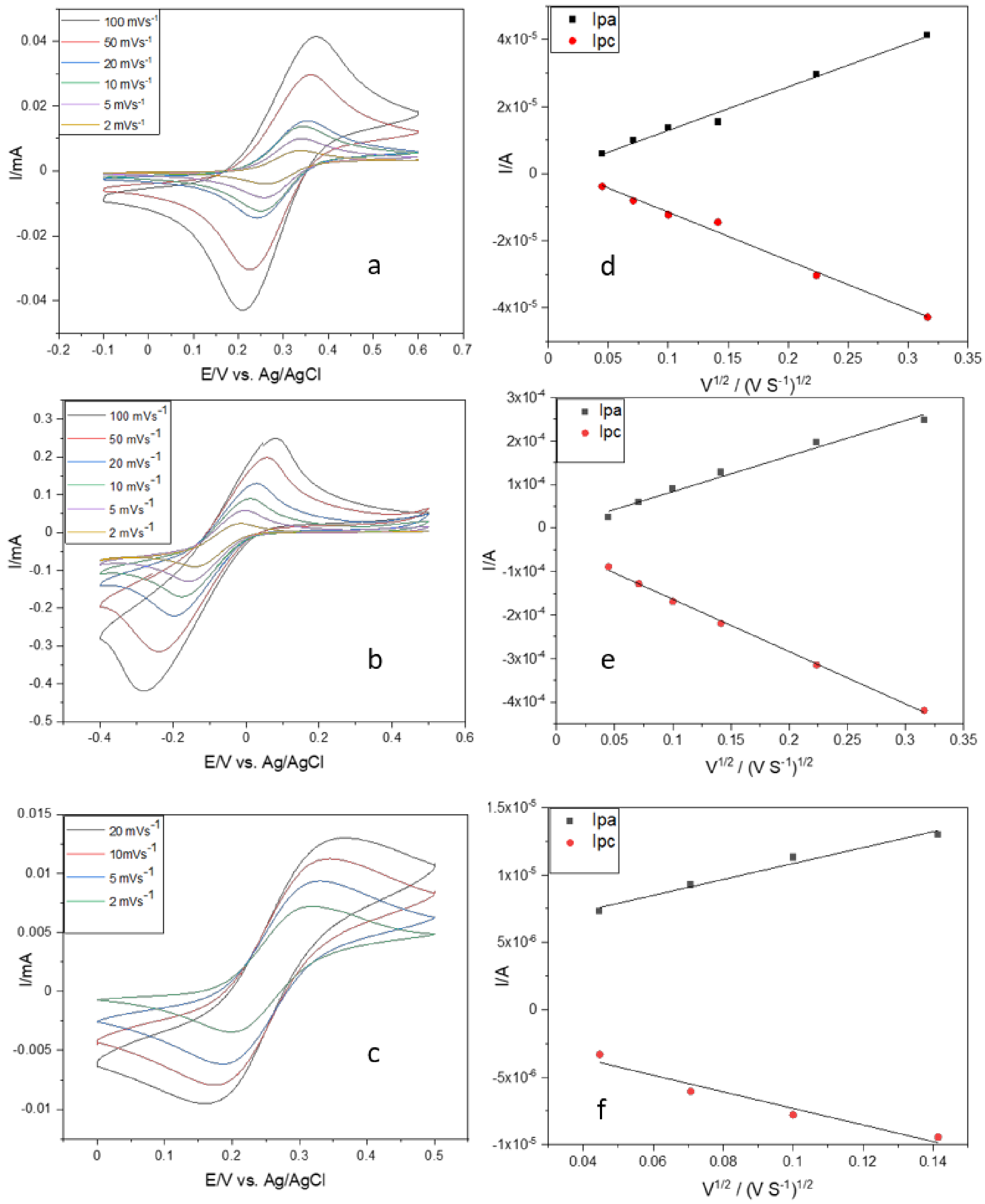

Figure S19. The CV's of (a) 0.5 mmol dm^{-3} [FcMeOH], (b) 5 mmol dm^{-3} $[Ru(NH_3)_6]^{3+/2+}$ and (c) 0.5 mmol dm^{-3} $[Fe(CN)_6]^{4-/3-}$ at various scan rates (2, 5, 10, 20, 50 and 100 mV s^{-1} for [FcMeOH] and $[Ru(NH_3)_6]^{3+/2+}$ redox species and 2, 5, 10 and 20 mVs^{-1} for $[Fe(CN)_6]^{4-/3-}$ redox couple); reliance of peak current as a function of square root of scan rates for the film containing C_{16}TAB deposited at -1.25 V (vs. Ag/AgCl) for 20 s on an ITO electrode. All experiments were carried out after surfactant removal: (d) [FcMeOH], (e) $[Ru(NH_3)_6]^{3+/2+}$ and (f) $[Fe(CN)_6]^{4-/3-}$.

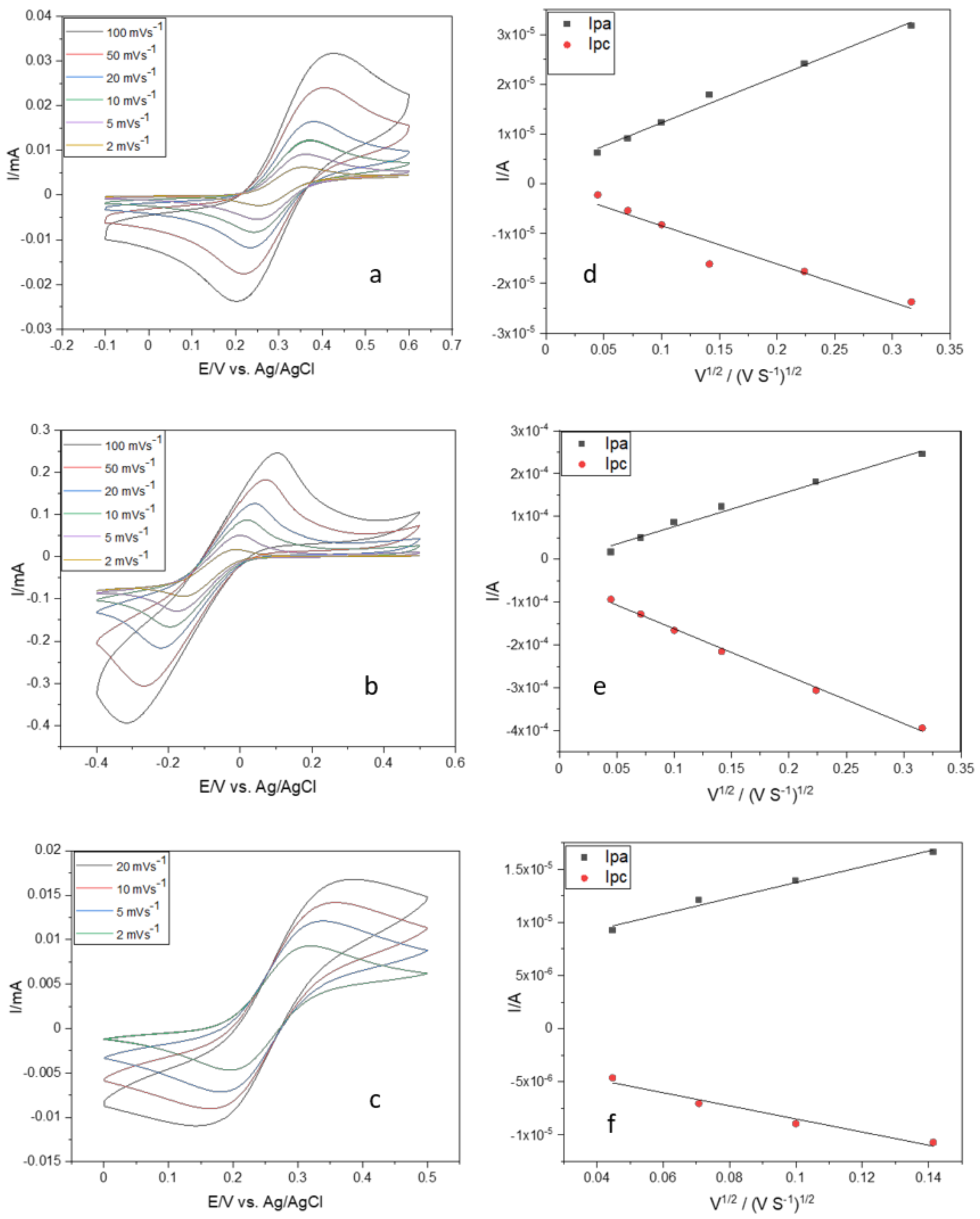

Figure S20. The CV's of (a) 0.5 mmol dm^{-3} [FcMeOH], (b) 5 mmol dm^{-3} $[Ru(NH_3)_6]^{3+/2+}$ and (c) 0.5 mmol dm^{-3} $[Fe(CN)_6]^{4-/3-}$ at various scan rates (2, 5, 10, 20, 50 and 100 mV s^{-1} for [FcMeOH] and $[Ru(NH_3)_6]^{3+/2+}$ redox species and 2, 5, 10 and 20 mVs^{-1} for $[Fe(CN)_6]^{4-/3-}$ redox couple); reliance of peak current as a function of square root of scan rates for the film containing C_{18}TAB deposited at -1.25 V (vs. Ag/AgCl) for 20 s on an ITO electrode. All experiments were carried out after surfactant removal: (d) [FcMeOH], (e) $[Ru(NH_3)_6]^{3+/2+}$ and (f) $[Fe(CN)_6]^{4-/3-}$.

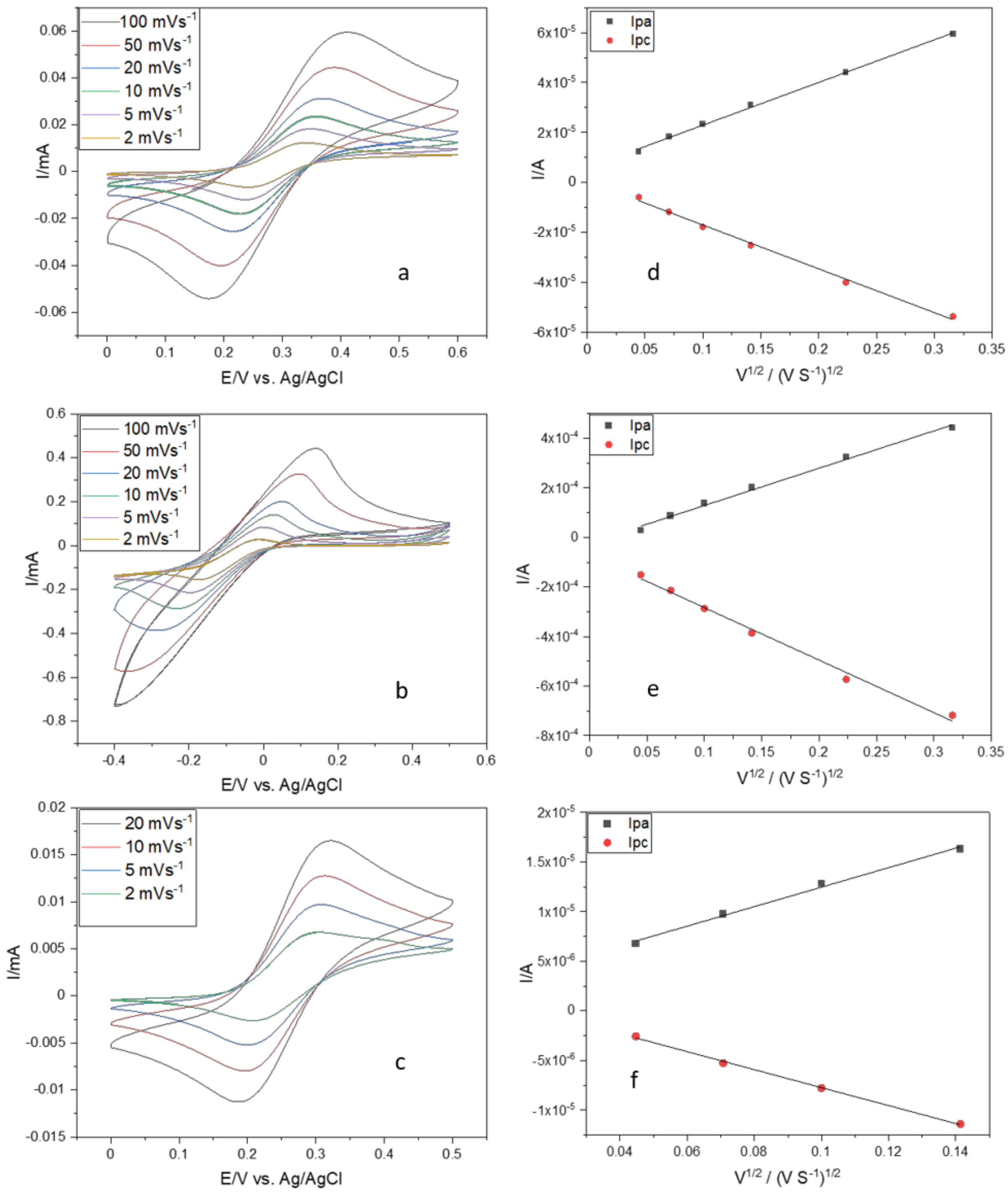

Figure S21. The CV's of (a) 0.5 mmol dm^{-3} [FcMeOH], (b) 5 mmol dm^{-3} $[Ru(NH_3)_6]^{3+/2+}$ and (c) 0.5 mmol dm^{-3} $[Fe(CN)_6]^{4-/3-}$ at various scan rates (2, 5, 10, 20, 50 and 100 mV s^{-1} for [FcMeOH] and $[Ru(NH_3)_6]^{3+/2+}$ redox species and 2, 5, 10 and 20 mVs^{-1} for $[Fe(CN)_6]^{4-/3-}$ redox couple); reliance of peak current as a function of square root of scan rates for the film containing C_{20}TAB deposited at -1.25 V (vs. Ag/AgCl) for 20 s on an ITO electrode. All experiments were carried out after surfactant removal: (d) [FcMeOH], (e) $[Ru(NH_3)_6]^{3+/2+}$ and (f) $[Fe(CN)_6]^{4-/3-}$.

Table S1. The porosity values (φ) determined from the pore diameter and pore spacing measurements.

Surfactants	Pore diameter / nm	Pore area / nm^2	Spacing/ nm	Unit cell area / nm^2	Porosity (fraction)
C_{14}	1.3	1.3	4.0	13.8	0.1
C_{16}	2.8	6.2	4.4	16.5	0.4
C_{18}	3.2	8.2	4.8	19.9	0.4
C_{20}	3.8	11.5	5.1	22.1	0.5
C_{22}	4.4	15.2	5.1	22.5	0.7
